# Cold Climate Impact on Air-Pollution-Related Health Outcomes: A Scoping Review

**DOI:** 10.3390/ijerph19031473

**Published:** 2022-01-28

**Authors:** Osnat Wine, Alvaro Osornio Vargas, Sandra M. Campbell, Vahid Hosseini, Charles Robert Koch, Mahdi Shahbakhti

**Affiliations:** 1 Department of Mechanical Engineering, Faculty of Engineering, University of Alberta, Edmonton, AB T6G 1H9, Canada; osnat@ualberta.ca (O.W.); bob.koch@ualberta.ca (C.R.K.); 2 Department of Paediatrics, Faculty of Medicine & Dentistry, University of Alberta, Edmonton, AB T6G 1C9, Canada; osornio@ualberta.ca; 3 Health Sciences Library, University of Alberta, Edmonton, AB T6G 2R7, Canada; scampbel@ualberta.ca; 4 School of Sustainable Energy Engineering, Simon Fraser University, Surrey, BC V3T 0N1, Canada; vahid_hosseini@sfu.ca

**Keywords:** traffic-related air pollution (TRAP), vehicle emissions, cold climate, vehicle cold start, low temperatures, human health, transportation, climate change, extreme weather

## Abstract

In cold temperatures, vehicles idle more, have high cold-start emissions including greenhouse gases, and have less effective exhaust filtration systems, which can cause up to ten-fold more harmful vehicular emissions. Only a few vehicle technologies have been tested for emissions below −7 °C (20 °F). Four-hundred-million people living in cities with sub-zero temperatures may be impacted. We conducted a scoping review to identify the existing knowledge about air-pollution-related health outcomes in a cold climate, and pinpoint any research gaps. Of 1019 papers identified, 76 were selected for review. The papers described short-term health impacts associated with air pollutants. However, most papers removed the possible direct effect of temperature on pollution and health by adjusting for temperature. Only eight papers formally explored the modifying effect of temperatures. Five studies identified how extreme cold and warm temperatures aggravated mortality/morbidity associated with ozone, particles, and carbon-monoxide. The other three found no health associations with tested pollutants and temperature. Additionally, in most papers, emissions could not be attributed solely to traffic. In conclusion, evidence on the relationship between cold temperatures, traffic-related pollution, and related health outcomes is lacking. Therefore, targeted research is required to guide vehicle regulations, assess extreme weather-related risks in the context of climate change, and inform public health interventions.

## 1. Introduction

Outdoor air pollution has long been recognized as a threat to human health, contributing to mortality and morbidity, and it is expected to be worsened by extreme weather events related to climate change. Several body systems are impacted, from acute short-term outcomes to chronic long-term health effects, such as the respiratory and cardiovascular systems, or fetal development during pregnancy [1]. The World Health Organization declared that ambient air pollution is a significant environmental risk to health and mortality [2]. In addition, the International Agency for Research on Cancer considers outdoor air pollution a leading environmental cause of cancer deaths [3]. In most cities, transportation is a significant source of air pollution leading, for example, to early deaths in the US [4].

Traffic contributes to a wide range of short- and long-lived air pollutants, including primary pollutants (particulate matter, nitrogen oxides, sulphur oxides, carbon monoxide, volatile organic compounds, and unburned hydrocarbons), and secondary pollutants (ozone, particulate matter, and secondary organic aerosols (SOA)). Transportation contribution to air pollution is from the combustion engine and vehicle tailpipe emissions, exhaust, tire and brake wear, road resuspended dust particles, and evaporative emissions. Zero-emission clean vehicle technologies, such as fuel cell electric vehicles and electric vehicles, still generate wear and resuspended dust particles [5,6]. A vehicle generates emissions when it is at the beginning of the trip (cold start emissions), during the trip (hot exhaust and resuspended dust particles), idling, and at the end of the trip when the vehicle is parked (evaporative emissions). All modern on-road and off-road vehicles contain exhaust aftertreatment (i.e., filtration) systems that contribute to lowering criteria air contaminants. However, suppose the vehicle exhaust aftertreatment system is not operating in its design conditions (mainly temperature-related); in that case, it may emit different pollutants such as unburned hydrocarbons and CO, or NO_2_ and NH_3_, which can contribute to public health risks [7,8].

In addition, a cold climate adversely impacts vehicle energy consumption and greenhouse gas emissions, quantified by total-cycle CO_2_ emissions. Zero-emission and low-emission vehicle technologies, such as electric vehicles and plug-in hybrid electric vehicles (PHEV), are not exempt. The electric range is reduced, and emissions (PHEV) are increased [9]. There is a strong regional-dependency on battery electric vehicle (BEV) performance (reduced electric range, increased CO_2_) [10,11].

The exhaust of a vehicle contains many toxic substances, for which only four main components are regulated: carbon monoxide (CO), unburned hydrocarbons (HC), nitrogen oxides (NOx), and particulate matter (PM). Many other classes of toxic material are included in the four main categories, but are not measured separately. For example, HC consists of a wide range of hydrocarbons that may consist of polycyclic aromatic hydrocarbons (PAHs); a few are known carcinogens. NOx (i.e., NO, and NO_2_) has a different health impact, and the NOx fraction may change significantly depending on vehicle technology. PM contains sulphate and nitrate, organic matter, metals (engine lube oil and engine wear), and black carbon. PM is an indicator of the total mass of particles emitted from the exhaust. Still, particle size and particle number (PN) in the nano-size range are significantly more important when new engine technologies are used. PN emission limits have only recently been introduced in a limited European class of vehicles.

Another documented issue with vehicular emissions is that in-use emissions are significantly higher than certification limits, due to lack of maintenance, fuel and lube oil quality, exhaust tampering, and adverse weather conditions such as cold climate. In many cases, emissions are sacrificed for power and fuel economy [12,13]. Additionally, a cold climate can impact vehicle emissions’ characteristics and, consequently, air quality. Reducing harmful exhaust gas emissions in conventional vehicles relies on exhaust aftertreatment systems, which require operating temperatures of 300 °C to 400 °C, unavailable during the initial cold phase of vehicle operation. For example, during a cold start, the temperature of the particulate filter is low, which is further intensified under a cold climate, since it will take longer for the particulate filter to reach the required operating temperature [14,15]. Thus, PM emissions from the engine directly go to the environment.

Moreover, the duration of the cold phase period in a vehicle increases substantially under cold temperatures. In cold climate cities, most urban short trips (e.g., 10–15 min) conclude before the vehicle has reached a fully warmed-up condition. For instance, on a cold winter day, most vehicles, after traveling a short distance to a school drop-off zone, are still in the vehicle cold phase with the low-efficiency operation of the exhaust aftertreatment systems.

Thus, harmful vehicular emissions drastically increase as vehicle start-up temperature decreases [16]. In contrast, existing emission regulations only consider vehicles’ tailpipe emission and operating temperature down to −7 °C [17], well above many countries’ cold season temperatures. The −7 °C cold testing requirement is not even across all jurisdictions and vehicle technologies. Hence, it is expected that engine control unit calibrations are not tuned to maximize emission reduction below that temperature limit. Furthermore, the levels of air pollution in below-zero climates may also be intensified by house heating combustion processes and meteorological conditions, such as thermal inversions, which trap and increase pollutant levels. Thus, extreme cold (or hot) temperatures can influence pollutant levels in the air, and their chemical mixtures [18]. These conditions may affect public exposure to harmful vehicular emissions in cold temperatures, increasing health risks, decreasing productivity, and impacting climate change.

A recent study on the effects of temperatures on mortality shows that cold temperatures have more consequences than hot temperatures, without considering the role of air pollution [19]. While many research studies have identified the impact of warm temperatures on air pollution-related health outcomes, there is scarce evidence addressing those links in a cold climate, i.e., below-zero and below −7 °C [20,21]. It is also unclear how much further prospective emission regulations should go for the sake of air pollution and public health, for example, considering coexisting variables such as ambient temperature [22].

This scoping review aims to map the scope and nature of the scientific literature on the impact of a cold-weather climate on traffic-related air pollution and the resulting health outcomes. This initiative is a collaboration between engineering and environmental health researchers, as part of a larger project funded by Canada’s SSHRC Knowledge Synthesis Grants in the area of “Living within the Earth’s Carrying Capacity” [23]. We aim to learn about the breadth and depth of existing literature and identify the existing research gaps responding to the following questions:

- What are the reported relationships between health impacts and air pollution in cold weather (i.e., below zero °C temperatures)?
- Does cold weather modify the health impacts of air pollution?
- Are the health impacts of air pollution reported in cold temperature different from those identified in warm temperature?
- Can the health effects in cold climates be directly attributed to a specific source, e.g., traffic?

Enhancing the understanding of the potential health risks of traffic-related air pollution in a cold climate and disseminating the results could help identify areas of opportunity to minimize pollution’s impact on health in cold climate countries and locations impacted by harsh winters due to climate change. Moreover, it will support future research to fill the identified research gaps.

## 2. Materials and Methods

The scoping review followed the Joanna Briggs Institute (JBI) guidelines for scoping reviews [24], and the frameworks developed by Arksey and O’Malley and Levac et al. [25,26]. The protocol for this study has been published elsewhere [27]. Predefined inclusion and exclusion criteria guided the search strategies and the decisions on which data sources will be included (Table 1). The following elements of *Population*, *Concept* and *Context* (as described by the JBI manual) guided the inclusion criteria:

*Population:* Human health outcomes with no restrictions on age or gender;

*Concept:* Traffic-related air pollution in cold climates;

*Context:* Cold climate, i.e., locations with below-zero temperatures in the cold season.

### 2.1. Identifying the Research Question

The scope of the inquiry was guided by the research questions to identify what is known about the impact of cold climate on air pollution-associated health outcomes.

### 2.2. Identifying Relevant Studies

An initial search in several search engines was undertaken by an expert searcher librarian (SC) to identify articles on the topic. The text words contained in the titles and abstracts of relevant articles, and the index terms used to describe the papers, were used to develop a complete and refined search strategy from the following search engines: OVID Medline, Ovid EMBASE, SCOPUS, Proquest Dissertations and Theses Global, Compendex (Engineering Village), EBSCO Environment Complete, and PROSPERO using a controlled vocabulary (e.g., MeSH, Emtree) and keywords representing the concepts “automobiles,” “pollution,” “cold climate,” and “health effects.” Searches were adjusted appropriately for different databases. Additional citations from other sources were added for screening. Reference lists of included papers were screened for further relevant articles. Only studies published in English (following the team’s language proficiency) for which the full text was available until the end of 2020, were included [24].

### 2.3. Study Selection

**Figure 1:**
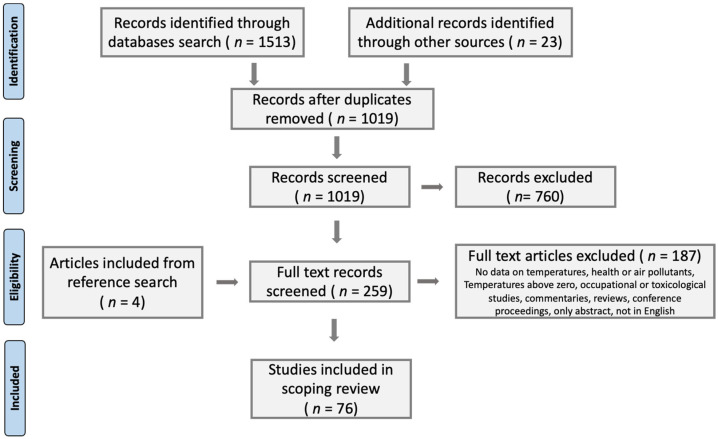
PRISMA flow diagram.

Citations were uploaded into RefWorks, and Covidence, where 517 duplicates were removed. One-thousand and nineteen titles and abstracts were screened by two independent reviewers (OW and AOV) against the inclusion criteria for the review, and irrelevant studies were removed. The full text of 259 papers was assessed in detail against the inclusion criteria by the reviewers. One-hundred and eighty-six papers were excluded. Reasons for exclusion included: no data on temperatures; outdoor pollution levels or health outcomes; temperature levels reported were above zero; occupational or toxicological studies; not in English; not peer-reviewed; or the full text was not available (e.g., meeting proceedings, abstracts). Four relevant papers were added through a snowball methodology. Any disagreements that arose between the reviewers at each stage of the selection process were resolved through discussion. The different phases of the search and the results can be seen in a PRISMA flow diagram [28] (Figure 1).

### 2.4. Charting the Data

Using a data extraction form developed by OW and AOV, data were extracted from the included papers (OW) and verified by a second reviewer (AOV). Data extraction categories were modified and revised as necessary during each included study’s iterative data extraction process. The data extracted included specific details on:

- Paper characteristics (authors, the years studied and published, study location);
- The participants (population studied);
- Concept (air pollution sources and assessment, pollutant studied and reported levels, health outcomes explored);
- Context (temperature values and seasons studied);
- Study design and methodology (methods, confounders, lag time);
- Key findings relevant to the review questions as declared by the authors of the papers.

### 2.5. Collating, Summarizing, and Reporting the Results

Data Analysis was conducted by two reviewers (OW and AOV). In addition, regular consultations took place throughout the iterative process of data extraction, collation, and analysis with the larger interdisciplinary team. The diverse composition of the authors’ team provided various perspectives on the topic, input, and guidance towards the subsequent phases of the literature review and helped identify the literature gaps. The findings are presented through qualitative descriptions, summary tables, and figures to map the data.

## 3. Results

We included 76 out of 1536 publications for the final evaluation, investigating the health impacts of air pollution in cold climate countries (Figure 1). A summary of the details of all identified papers is provided in Table S1.

The identified papers were published between 1996–2020, studying various time frames—the earliest from 1985 and the latest from 2016. Our search included all cold climate regions or countries. However, we identified studies only from the following countries: thirty-seven papers from North America (Canada *n* = 24, USA *n* = 13); twenty-three papers from Europe (UK *n* = 5, Germany *n* = 4, Sweden *n* = 4, Iceland *n* = 2, the Netherlands *n* = 2, one from each of the following countries, Croatia, Denmark, Lithuania, Norway, and Spain and one that explored several European cities); and sixteen papers from Asia. Sixty-six papers focused on the associations between air pollution and health (in this context, associations refer to a statistical relationship between an independent variable, i.e., air pollution in a cold climate and dependent one, i.e., health outcomes). Another ten articles focused on the effect of temperature on health outcomes.

The papers included several study designs, including time series, case-cross-over, comparative research, and ecological. Moreover, the studies used various methodologies and statistical approaches such as generalized linear mixed models, additive Poisson regression models, or general additive models.

Besides controlling for temperature in those papers that focused on the impact of air pollution, or controlling for air pollution in those exploring the temperature effects on health, many other confounders were included (e.g., SES, smoking) (Table S1). Sixty-one papers investigated lag effects of more than one day in their analysis. Only two papers explored longer-term outcomes (i.e., whole pregnancy or season in the following year).

### 3.1. Health Outcomes

The reviewed studies explored associations of one or several health outcomes with air pollution in a cold climate. Morbidity and mortality related to respiratory and cardiovascular effects were the most analyzed health outcomes. Specific health outcomes studied include the following: all-cause cardiovascular and respiratory mortality (*n* = 16), various respiratory diseases (COPD, asthma, bronchitis, nasal symptoms, wheeze, allergic sensitization) (*n* = 16), cardiovascular diseases (myocardial infarction, stroke, arrhythmia, cardiac autonomic function, acute coronary syndrome, ischemic heart disease) (*n* = 13), and mental health (depression, suicide attempts, substance use, various) (*n* = 7). In addition, other papers studied several health outcomes, including headaches, migraines, rheumatoid arthritis, abdominal pain, chest pain and weakness, conjunctivitis, epistaxis, appendicitis, birth outcomes, otitis media, collection of symptoms, ED admission, ambulance dispatches for various reasons, and testing of biomarkers of disease (glucose metabolism, systemic inflammation) (*n* = 16). Health data were obtained from registries on mortality, ER visits, hospitalizations, outpatient clinics, or self-reports. The reviewed studies examined the whole population (*n* = 47), only adults and seniors (*n* = 10), individuals with pre-existing diseases (*n* = 9), eight papers focused on children (1–18 years old), and another two papers focused mainly on children (1–20 and 8–24 years old).

### 3.2. Air Pollution

Twenty-eight articles described the source of air pollution as a combination of traffic indicators (i.e., specific pollutants) and other combustion sources, geologic elements, studded tires, or dust. Another 28 articles did not discuss any specific source for air pollution. Twenty articles indicated that the source of air pollution in the location studied was mainly traffic-related. The pollutants studied were related to traffic emissions (e.g., BC, NO_2_).

Data on air pollution was predominantly collected from monitoring stations (*n* = 72). Other air pollution exposure metrics used in a few papers included proximity to main roads or heavy traffic (*n* = 2), dispersion models (*n* = 2), satellite remote sensing (*n* = 1), and personal monitors (e.g., home, car) (*n* = 2) to characterize air pollution.

**Figure 2:**
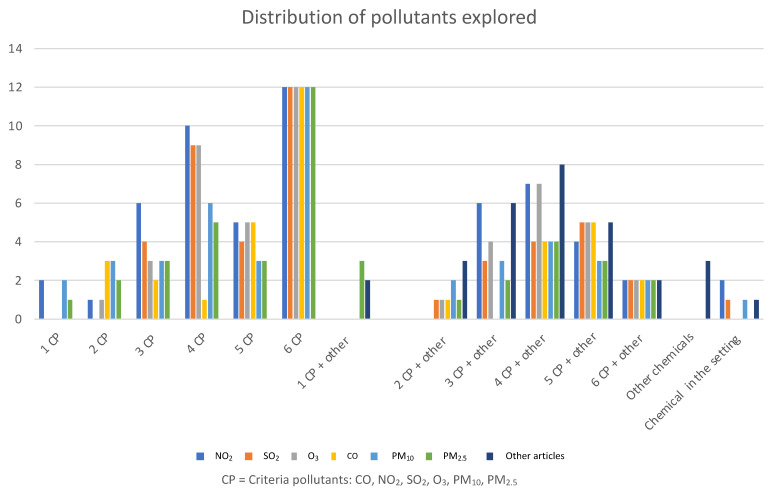
Distribution of the various pollutants studied in relation to health outcomes. 1 CP = the number of papers which explored one type of criteria pollutant. 2 CP–6 CP = the number of papers that explored multiple combinations of 2–6 criteria pollutants. Other = pollutants explored other than criteria pollutants including: Black carbon, black smoke, NO, NOx, NO_3_, SO_4_, sulphate H2S PAHs, COH, trace elements (copper, zinc, bromine, lead, iron, silicon, calcium, manganese, nickel, vanadium, selenium, sulphur, and potassium), volatile organic compounds (VOCs) (formaldehyde, benzene, toluene, methyl tert-butyl ether (MTBE)), particles, PNC (particulate number concentration/UFP indicator) or UFP, Delta-C, PM_2.5–10_, PN (particulate number), PNam (accumulation mode particle number), PM_2.5_ bound hopanes, PMion (sum of sulphate nitrate and ammonium), PMrest (the difference between urban PM_10_ and rural; a proxy for locally generated PM_10_), PM_2.5_ components, PM_<2.5_, AMP (accumulation mode).

Various chemicals were studied concerning health outcomes (Figure 2). Most studies (*n* = 45) explored one to six criteria pollutants (CO, NO_2_, SO_2_, O_3_, PM_10_, PM_2.5_) in different combinations. Twenty-six studies examined various other pollutants along with criteria pollutants. Three papers focused on non-criteria pollutants only (i.e., black carbon (BC), Delta-C, particulate number concentration (UFP), accumulation mode AMP, PM_<2.5_, Methyl tert-butyl ether (MTBE)). Two articles did not study pollutants but merely described the pollution level in the location where the study was conducted (Figure 2). Only one pollutant was studied in seven studies: PM_2.5_, PM_10_, MTBE, black carbon. Additional details on the pollutants tested in the reviewed papers are available in Table S2).

The studies analyzed pollutants individually, and not as a mixture. An air quality index characterized pollution (AQHI, API, AQI) in a few instances.

### 3.3. Cold Climate

As per our inclusion criteria, all the papers included in this review studied locations that reach below-zero temperatures. Forty-seven papers were from areas that registered ≤ −7 °C temperatures, and 29 papers were from areas that had temperatures between 0 °C and −7 °C. Some articles provided further stratification of the temperatures according to percentile distributions. Eight papers explored winter data only, but most of the studies included an average of all-year temperatures in their analysis (*n* = 68). Of those analyzing year-round data, thirty-eight papers also explored warm and cold seasons (*n* = 38), four seasons, and/or both warm and cold seasons (*n* = 6). Twenty-four papers did not include seasonality and only used yearly average data (*n* = 8).

### 3.4. Connecting Health Outcomes to Air Pollution in Cold Climate Locations

Most articles mainly focused on the relationship between pollution and health outcomes (*n* = 66) after adjusting for temperature. Ten papers focused on temperatures. Two papers adjusted for pollutants, and only eight papers explored the effect of temperatures on pollution levels and related health outcomes (interactions). Temperature and seasons were tested as variables in those eight studies.

#### 3.4.1. Air Pollution and Health Outcomes Associations in a Cold Climate Location

**Figure 3:**
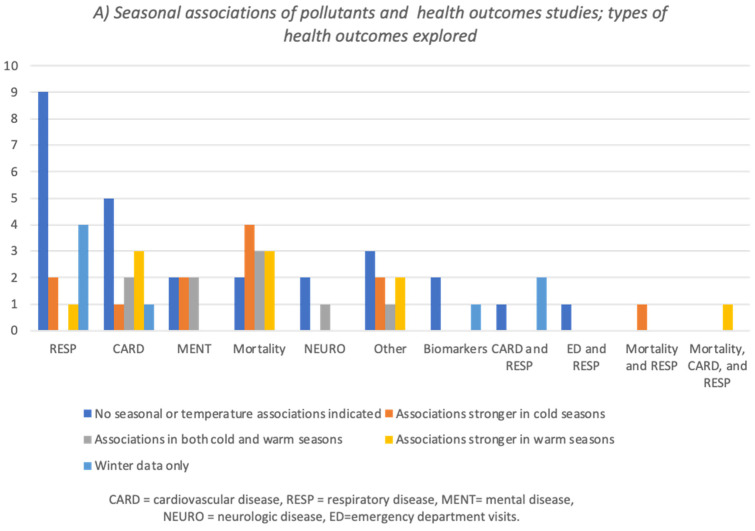

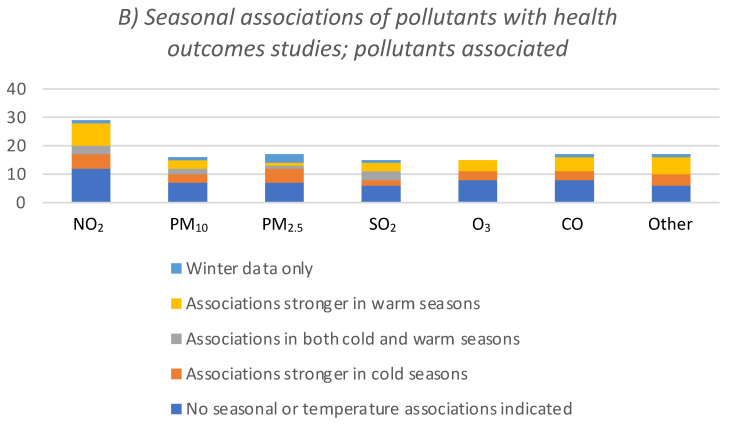

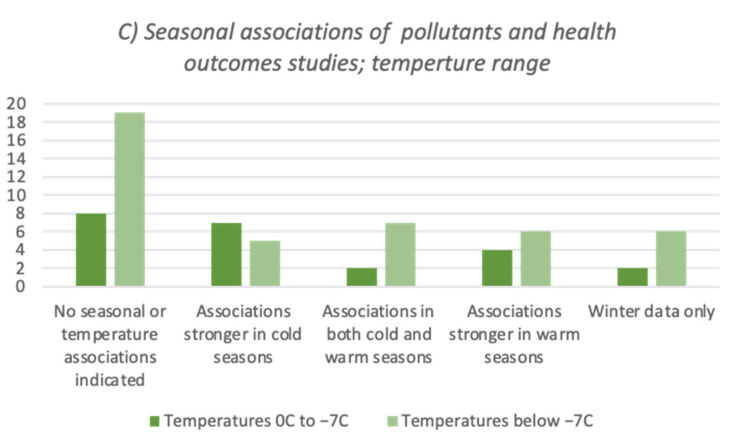
Pollution and health studies. Health outcomes with seasonal associations (**A**), the pollutants involved (**B**), and the temperature range reported (**C**). Legend: Blue = papers that used winter data only; yellow = papers that identified stronger associations with health outcomes in the warm seasons; gray = papers that identified strong associations with health outcomes in both warm and cold seasons; orange = papers that identified stronger associations with health outcomes in the cold season; blue = papers that did not identify seasonal associations with health outcomes; dark green = 0 °C to −7 °C; light green = below −7 °C.

Sixty-six papers explored the association between air pollution and various health outcomes in a cold climate. The studies differed in the statistically significant associations with different health outcomes, related or unrelated to seasons or temperature. Overall, among the pollutants identified as associated with health outcomes, NO_2_ and PMs were the most frequently involved (Figure 3). Additional details are available in Table S3.

1. Twenty-seven papers identified an association between air pollution and health outcomes with no indication of seasonal or temperature relation, including two papers that mentioned pollution levels as a contextual description [29,30,31,32,33,34,35,36,37,38,39,40,41,42,43,44,45,46,47,48,49,50,51,52,53,54,55].
2. Thirty-one papers indicated different seasonal associations between pollutants and health outcomes:
  (a) Twelve papers identified that the association between pollution and health outcomes was more robust in the cold seasons/temperatures [56,57,58,59,60,61,62,63,64,65,66,67];
  (b) Nine papers identified associations of pollution and health in both cold and warm seasons/temperatures [68,69,70,71,72,73,74,75,76];
  (c) Ten papers reported stronger associations in the warm temperature season [77,78,79,80,81,82,83,84,85,86].
3. Another eight papers explored only winter data [87,88,89,90,91,92,93,94]. In this subset of papers, the authors were interested in the possible role of cold weather in health outcomes or explaining alterations in body function and symptoms in response to winter conditions, related industrial emission trends, wood-burning smoke, pollutant transport, and inversions [87,92,93]. Others recognized existing differences in the pollutant mixtures from summer [89,92]. Finally, Stieb et al. [92] focused on winter data because it allowed the evaluation of multi-pollutant models and aggregated pollutant indices.

#### 3.4.2. Temperatures/Seasons and Health Outcomes

Two papers focused on the effects of temperature, including high and low temperatures. One study identified that extremely hot temperature was positively associated with all-cause and cardiovascular disease ambulance calls. In contrast, extremely cold temperatures were positively associated with all-cause, cardiovascular and respiratory morbidity [95]. The second paper identified warmer temperatures associated with adverse birth outcomes [96]. In these two papers, the analysis models adjusted for pollutants.

#### 3.4.3. Temperature/Season Impact on Air Pollution and Health Outcomes

**Figure 4:**
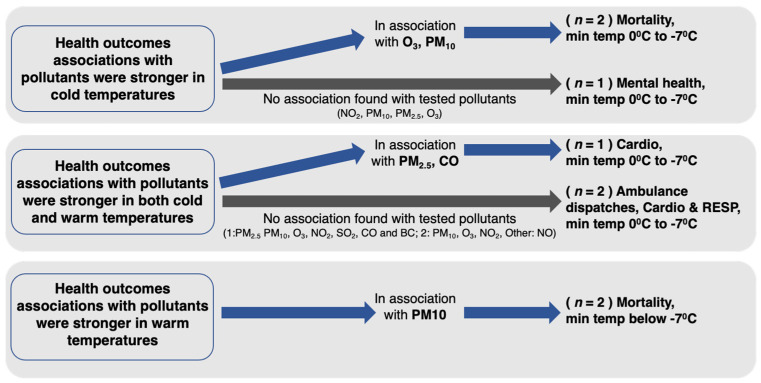
Eight studies tested the temperature and air pollution with health outcomes. Blue arrow = association with tested pollutants black arrow = no association with tested pollutants (cardio = cardiovascular effects, RESP = respiratory disease); warm weather refers to temperatures between 23.3–40.6 °C.

Only eight papers explored the interaction between temperatures and pollution and their effect on health outcomes (Figure 4).

Five of them specifically explored and identified temperature as an effect modifier of the pollution impact on health outcomes [21,97,98,99,100].

All these papers analyzed data from China. In addition, four of those papers examined mortality as the health outcome, and one paper studied cardiovascular effects.

Two of those papers identified a stronger association in cold temperatures, aggravating the effects of pollution on mortality [21,97]. In both studies, the minimum temperatures were between 0 °C to −7 °C. Chen et al. [97] tested the effects of O_3_, considering PM_10_, NO_2_ and SO_2_. They identified that O_3_ effects were more substantial in the low-temperature days of the cold season—those with temperatures below 18 °C had the most potent modification effect. Cheng and Kan [21] evaluated the effects of PM_10_, SO_2_, NO_2_, and O_3_, identifying a statistically significant interaction between PM_10_/O_3_, and extremely low temperatures for non-accidental and cause-specific mortality. There was no significant interaction with SO_2_ or NO_2_.

In contrast, two papers identified that warm temperatures strengthened the effects of pollution (PM_10_) when assessing mortality [98,99]. Meng et al. (2012) tested PM_10_ only and Li et al. (2011) tested PM_10_, NO_2_, and SO_2_.

The fifth paper explored CO and PM_2.5_ and identified that warm and cold temperatures modified the associations with cardiovascular outcomes to various extents [100]. Significant interactions were identified between PM_2.5_ in the cold and CO in the warm seasons.

The authors explored the effects of several pollutants and temperatures in the remaining three papers and their association with various outcomes (mental health, ambulance dispatches, and cardio-respiratory outcomes). Tested contaminants included NO_2_, PM_10_, PM_2.5_, O_3_, SO_2_, CO and NO, and the locations included recorded minimum temperatures between 0 °C and 7 °C. The health outcomes were associated with cold temperatures [101] and cold and warm temperatures [102,103], but not with the pollutants.

## 4. Discussion

We identified and reviewed 76 papers exploring the relationship between health outcomes and air pollution in cold temperatures (below 0 °C). The examined health outcomes were primarily short-term and predominantly centred on respiratory and cardiovascular morbi-mortality. The reviewed papers predominantly relied on criteria pollutants data. Most papers did not test for interactions of temperature, air pollution, and health outcomes. Rather they aimed to understand the relationship between pollution and health or temperature and health, where temperature or pollutants were considered confounders. Some of these papers described seasonal differences but did not formally explore the interactions of the three variables of interest, except for eight articles. Five of them identified that both cold and warm temperatures impacted the magnitude of O_3_, PM_2.5_, PM_10_, and CO effects on increased mortality and cardiovascular diseases when combined. Three of the eight papers reported associations of health outcomes and pollutants with cold temperatures in locations that did not reach temperatures lower than −7 °C. Another three studies found no association with the contaminants tested since only health outcomes and low temperatures showed associations. Hence, the findings provide a limited understanding of cold temperatures’ impact on traffic pollution and health outcomes, identifying that extreme temperatures potentiate air pollution health effects—a relevant aspect of climate change.

### 4.1. The Impact of Cold Temperatures on Pollution and Health

Low and high temperatures can impact the pollution mixture, pollutant levels, and related health outcomes [99,104]; however, in most studies exploring health outcomes, the temperature or pollution are adjusted as a confounder to avoid potential bias to the research analysis and findings [99]. Some of the studies reviewed suggest a possible association of seasonality with pollution-related health outcomes, which aligns with other reports [105]. However, in the reviewed papers, most studies considered temperature a confounder; therefore, temperatures were adjusted in the analysis models. Thus, they removed the possible relationship with temperature. The evidence is even more scarce when considering cold temperatures below −7 °C, as in many cities in the world. Harsh winters, and the spectrum of cities where climate has changed (e.g., temperatures in Texas, USA, dropped to −17 °C in February 2021) are attributed to climate change [106,107], and their possible impacts on pollution and health bring further attention to this topic. While the effects of warm weather are likely better documented, there is limited evidence about the effects of cold temperatures, which invites a targeted exploration in cold locations to understand health impacts and pollution [21]. This is a gap in our understanding of the association between cold temperatures, air pollution and related population health. Studying the impact of cold temperatures on pollution and identifying health outcomes is a complex question that challenges existing methodologies. Available epidemiological methods can provide information on the seasonality of disease and pollution. However, bringing these together is more challenging and requires novel research methods, e.g., artificial intelligence.

### 4.2. Air Pollutants in a Cold Climate

Although we focused on traffic-related air pollution, it was impossible to identify papers that exclusively used traffic-related pollution data. Most studies used monitored data, which is readily available for research purposes. However, monitoring stations capture pollutants from all sources, making the direct connection between traffic pollution and health uncertain.

More specific connections could be made with better exposure characterization. NO_2_ and PMs were most frequently associated with health outcomes in the cold season among the criteria pollutants studied. Both contaminants (NO_2_ and PMs) are commonly referred to as indicators of traffic pollution. However, they can also originate from other sources. The few papers identified by this review that explored temperature’s impact suggest the need to examine its role further when assessing PMs-, O_3_-, and CO-related health impacts [21,97,100]. Existing literature identified that pollutants behave differently according to seasons and regions. Therefore, this invites future research to determine the interactive effects between temperatures and pollution while considering geographical variations [20,105].

Apart from criteria pollutants, the papers reviewed tested other pollutants. Indeed, criteria pollutants are monitored because of their known potential health impact. However, while there is substantial evidence on criteria pollutants, there are other pollutants we know little about, which may significantly affect health outcomes. For example, gasoline direct injection (GDI) technology introduced for improved fuel economy emits nanoparticles due to mixing direct injection jet of fuel into the air [108]. SCRs (selective catalytic reduction) use a toxic agent, NH_3_ (ammonia), as a reducing agent for NOx conversion. However, NH_3_ may only be partially converted and released into the exhaust under conditions such as cold exhaust in a cold climate [109,110]. Usually, testing non-criteria pollutants implies limited monitoring based on complicated and expensive processes. This limits our understanding of various emission sources over time. The literature that explores other pollutants is growing. However, there is still a need to find ways to increase our knowledge of these pollutants (e.g., developing standardized methods, a shift in the conceptualization of air pollution beyond criteria pollutants, considering mixtures, increasing funding).

Thus, it is unclear whether or how cold climate may impact traffic emissions, composition and the atmospheric behaviour of pollutants, and the potential health impacts. The knowledge gap also increases because fuel composition is different in the winter and summer, affecting the type and mixture of emissions, a fact not noted in any of the papers identified for this review (except one article that explored MTBE use in the winter, which is no longer used). Studies show changes in the composition may impact ozone levels. This aligns with some of the findings identified in our review [21,97]. Additionally, there has been limited evidence in the papers reviewed on the impact of air pollutants in combination as mixtures; instead, most papers studied the individual pollutants’ role.

### 4.3. Health Outcomes Associations

The health outcomes studied in the reviewed literature included a variety of diseases and symptoms. These health outcomes reflect previously reported pollution-related effects. Some of the health outcomes reported in this review were directly associated with the cold season, implying the seasonality of diseases and increased health vulnerability in the cold season. A systematic review [105] identified that seasons modify the short-term effects of air pollution on morbidity. Although our review also found evidence of seasonal effects, we could not identify specific patterns of the associations between warm or cold seasons, pollutants, and health outcomes. Primarily, papers identified seasonal relationships after “removing” the effect of temperature (as indicated above), suggesting that seasonal exposures and measured effects involve the participation of other co-existing characteristics besides the temperature. For example, seasonal variations in the pollution chemical mixture composition, seasonal changes in the pollutants’ concentrations resulting from altered emissions and meteorological conditions, or human behaviour changes. This represents a clear gap in our understanding of the impact of cold temperatures on pollution and the related health outcomes.

### 4.4. Strengths and Limitations

A strength of this study is the multidisciplinary perspective brought by the team to include various perspectives into the review process and the identification of research gaps that have implications for car regulations, warning systems to vulnerable populations in specific weather conditions and locations, and public health.

The health outcomes reported in the reviewed literature primarily represent short-term impacts, and there is no evidence reflecting the long-term effects of relative exposure to low pollution levels. Therefore, our review could not capture the effects on chronic diseases, such as cancer and neurodevelopmental disorders.

It is possible that although we used several databases, supplemented the search with citation chaining, and manually searched recent years of relevant journals, some related articles on this complex topic may be missing.

## 5. Conclusions

Existing evidence on the relationship between cold temperatures, pollution, and adverse health outcomes is limited. The reviewed studies described various short-term health outcomes, some of which found seasonal associations of air pollution with morbidity and mortality, but mostly removed the possible impact of temperature from the findings. Only eight papers formally explored the complex modifying effect of temperatures on pollution levels and the resulting health outcomes. A broad exploration of the literature was necessary to identify the exitance of sufficient publications for more sophisticated reviews. Our results indicate there is not enough evidence to move in that direction.

Cold temperatures may contribute to a substantial increase in harmful vehicular tailpipe emissions. Air pollutants’ potential health impact in cold climate challenges current vehicle regulations.

Considering the lack of evidence on the impact of extremely cold temperatures on traffic-related air pollution and adverse health outcomes, the following gaps in the current literature are noted:

- Limited evidence on pollutants’ behaviour in a cold climate below −7 °C and their impact on health;
- Cold temperatures were often included as a confounder in the analysis, limiting our understanding of the combined impact of pollution and health;
- Limited knowledge on non-criteria pollutants and their impact on health in a cold climate;
- Minimal evidence explicitly addresses the impact of traffic-related emissions, cold temperatures, and health;
- Limited literature evaluating the long-term health effects of pollutants in a cold climate.

These identified gaps invite future interdisciplinary research that will employ advanced research methods to address the complexity of understanding mixtures of multiple pollutants and their health impact in a cold climate. This research will have implications for policy, regulations, and practice. In addition, it will guide which measures can be taken to mitigate the effects of traffic-related air pollution on health outcomes in a cold climate. Furthermore, there is a need to study the contribution of vehicle emissions in light of cold temperatures due to global climate change.

## Figures and Tables

**Table 1:** Inclusion and exclusion criteria.

	Inclusion	Exclusion
Paper characteristics	Any paper up to December 2020 Peer-reviewed (including reports) Full text in English	Abstracts from meeting proceedings. Not in English
Health outcomes	Human health only Any measurable health impact, including biomarkers of disease	Toxicology studies Risk assessment studies Exposure studies (i.e., chemical blood levels, computational estimated risk)
Pollution	Measurable urban outdoor air pollution	Indoor pollution Occupational exposures Emissions explicitly from sources other than traffic (e.g., fire, industry).
Cold Climate	Below-zero temperatures (e.g., minimum, average, mean, lower quartile) Measured temperature (range or average is indicated)	No measured temperatures provided

## Data Availability

The data presented in this study are available in the supplemantry material (Tables S1–S3).

## Supplementary Materials

The following supporting information can be downloaded at: https://www.mdpi.com/article/10.3390/ijerph19031473/s1, Table S1. Details of all reviewed papers. Table S2. The complete data on the pollutants tested in the papers reviewed (no pattern of specific pollutants tested at the same study was found). Table S3. The complete data on the papers which studied seasonal associations with health outcomes: types of health outcomes, pollutants associated, and temperature range indicated (no pattern identified).

## References

[1] Kampa M., Castanas E. (2008). Human health effects of air pollution. Environ. Pollut..

[2] World Health Organization (2018). Ambient (Outdoor) Air Pollution. https://www.who.int/news-room/fact-sheets/detail/ambient-(outdoor)-air-quality-and-health.

[3] World Health Organization International Agency for Research on Cancer (2015). IARC Monographs on the Evaluation of Carcinogenic Risks to Humans: Outdoor Air Pollution.

[4] Caiazzo F., Ashok A., Waitz I.A., Yim S.H.L., Barrett S.R. (2013). Air pollution and early deaths in the United States. Part I: Quantifying the impact of major sectors in 2005. Atmos. Environ..

[5] Amato F., Favez O., Pandolfi M., Alastuey A., Querol X., Moukhtar S., Bruge B., Verlhac S., Orza J., Bonnaire N. (2016). Traffic induced particle resuspension in Paris: Emission factors and source contributions. Atmos. Environ..

[6] Clougherty J.E., Kheirbek I., Eisl H.M., Ross Z., Pezeshki G., Gorczynski J.E., Johnson S.C., Markowitz S., Kass D., Matte T.D. (2013). Intra-urban spatial variability in wintertime street-level concentrations of multiple combustion-related air pollutants: The New York City Community Air Survey (NYCCAS). J. Expo. Sci. Environ. Epidemiol..

[7] Beckerman B.S., Jerrett M., Finkelstein M., Kanaroglou P., Brook J.R., Arain M.A., Sears M.R., Stieb D., Balmes J., Chapman K. (2012). The Association Between Chronic Exposure to Traffic-Related Air Pollution and Ischemic Heart Disease. J. Toxicol. Environ. Health Part A.

[8] Chen H., Goldberg M.S., Burnett R., Jerret M., Wheeler A., Villeneuve P. (2010). Long-Term Exposure to Traffic-Related Air Pollution and Cardiovascular Mortality. Epidemiology.

[9] Suarez-Bertoa R., Pavlovic J., Trentadue G., Otura-Garcia M., Tansini A., Ciuffo B., Astorga C. (2019). Effect of Low Ambient Temperature on Emissions and Electric Range of Plug-In Hybrid Electric Vehicles. ACS Omega.

[10] Wu D., Guo F., Field F.R., de Kleine R., Kim H.C., Wallington T.J., Kirchain R.E. (2019). Regional Heterogeneity in the Emissions Benefits of Electrified and Lightweighted Light-Duty Vehicles. Environ. Sci. Technol..

[11] Yuksel T., Michalek J. (2015). Effects of Regional Temperature on Electric Vehicle Efficiency, Range, and Emissions in the United States. Environ. Sci. Technol..

[12] Auto Express Thousands of UK Motorists Removing Diesel Particulate Filters. https://www.autoexpress.co.uk/car-news/consumer-news/95410/thousands-of-uk-motorists-removing-diesel-particulate-filters.

[13] Revolution Motors Enhance Your Diesel’s Performance & Efficiency with a DPF, DEF, or EGR Delete Service. https://revolutionmotors.ca/diesel-repair/egr-and-dpf-delete/.

[14] DieselNET Catalytic Diesel Filters. https://dieselnet.com/tech/dpf_catalytic.php.

[15] Mahadevan B.S., Johnson J.H., Shahbakhti M. (2015). Experimental and Simulation Analysis of Temperature and Particulate Matter Distribution for a Catalyzed Diesel Particulate Filter. Emiss. Control. Sci. Technol..

[16] Sakunthalai R.A., Xu H., Liu D., Tian J., Wyszynski M., Piaszyk J. Impact of Cold Ambient Conditions on Cold Start and Idle Emissions from Diesel Engines. Proceedings of the SAE Technical Paper Series, SAE International.

[17] European Union O.J. of the E.U. Regulation (EC) No 715/2007 of the European Parliament and of the Council. https://eur-lex.europa.eu/legal-content/EN/ALL/?uri=CELEX%3A32007R0715.

[18] Dominici F., Peng R.D., Barr C.D., Bell M. (2010). Protecting Human Health from Air Pollution. Epidemiology.

[19] Dear K., Wang Z. (2015). Climate and health: Mortality attributable to heat and cold. Lancet.

[20] Li J., Woodward A., Hou X.-Y., Zhu T., Zhang J., Brown H., Yang J., Qin R., Gao J., Gu S. (2017). Modification of the effects of air pollutants on mortality by temperature: A systematic review and meta-analysis. Sci. Total Environ..

[21] Cheng Y., Kan H. (2012). Effect of the Interaction Between Outdoor Air Pollution and Extreme Temperature on Daily Mortality in Shanghai, China. J. Epidemiol..

[22] Winkler S.L., Anderson J.E., Garza L., Ruona W.C., Vogt R., Wallington T.J. (2018). Vehicle criteria pollutant (PM, NOx, CO, HCs) emissions: How low should we go?. NPJ Clim. Atmos. Sci..

[23] The Social Sciences and Humanities Research Council Knowledge Synthesis Grant: Living with Earth’s Capacity. https://www.sshrc-crsh.gc.ca/funding-financement/programs-programmes/ksg_living_within_earth_carrying_capacity-ssc_vie_fonction_capacite_limite_terre-eng.aspx.

[24] Peters M., Godfrey C., McInerney P., Munn Z., Trico A., Khalil H. (2017). Chapter 11: Scoping Reviews. Joanna Briggs Inst. Rev. Man..

[25] Arksey H., O’Malley L. (2005). Scoping studies: Towards a methodological framework. Int. J. Soc. Res. Methodol..

[26] Levac D., Colquhoun H., O’Brien K.K. (2010). Scoping studies: Advancing the methodology. Implement. Sci..

[27] Wine O., Campbell S., Shahbakhti M., Hosseini V., Osornio Vargas A. (2021). Cold climate impact on air pollution related health outcomes: A scoping review protocol. Nat. Commun..

[28] Moher D., Tetzlaff J., Tricco A.C., Sampson M., Altman D.G. (2007). Epidemiology and Reporting Characteristics of Systematic Reviews. PLoS Med..

[29] Andersson M., Modig L., Hedman L., Forsberg B., Rönmark E. (2011). Heavy vehicle traffic is related to wheeze among schoolchildren: A population-based study in an area with low traffic flows. Environ. Health.

[30] Bremner S.A., Anderson H.R., Atkinson R.W., McMichael A.J., Strachan D.P., Bland J.M., Bower J.S. (1999). Short-term associations between outdoor air pollution and mortality in London 1992-4. Occup. Environ. Med..

[31] Carlsen H.K., Forsberg B., Meister K., Gíslason T., Oudin A. (2013). Ozone is associated with cardiopulmonary and stroke emergency hospital visits in Reykjavík, Iceland 2003–2009. Environ. Health.

[32] Chimonas M.-A.R., Gessner B.D. (2007). Airborne particulate matter from primarily geologic, non-industrial sources at levels below National Ambient Air Quality Standards is associated with outpatient visits for asthma and quick-relief medication prescriptions among children less than 20 years old enrolled in Medicaid in Anchorage, Alaska. Environ. Res..

[33] Dales R., Lee D.S., Wang X., Cakmak S., Szyszkowicz M., Shutt R., Birnie D. (2020). Do acute changes in ambient air pollution increase the risk of potentially fatal cardiac arrhythmias in patients with implantable cardioverter defibrillators?. Environ. Health.

[34] Dockery D.W., Luttmann-Gibson H., Rich D.Q., Link M.S., Schwartz J.D., Gold D.R., Koutrakis P., Verrier R.L., Mittleman M.A. (2005). Particulate air pollution and nonfatal cardiac events. Part II. Association of air pollution with confirmed arrhythmias recorded by implanted defibrillators. Res. Rep. (Health Eff. Inst.).

[35] Fung K.Y., Luginaah I., Gorey K., Webster G. (2005). Air Pollution and Daily Hospital Admissions for Cardiovascular Diseases in Windsor, Ontario. Can. J. Public Health.

[36] Grady S.T., Koutrakis P., Hart J.E., Coull B.A., Schwartz J., Laden F., Zhang J., Gong J., Moy M.L., Garshick E. (2018). Indoor black carbon of outdoor origin and oxidative stress biomarkers in patients with chronic obstructive pulmonary disease. Environ. Int..

[37] Hagen J.A., Nafstad P., Skrondal A., Bjørkly S., Magnus P. (2000). Associations between Outdoor Air Pollutants and Hospitalization for Respiratory Diseases. Epidemiology.

[38] Hertel S., Viehmann A., Moebus S., Mann K., Bröcker-Preuss M., Möhlenkamp S., Nonnemacher M., Erbel R., Jakobs H., Memmesheimer M. (2010). Influence of short-term exposure to ultrafine and fine particles on systemic inflammation. Eur. J. Epidemiol..

[39] Kaplan G.G., Szyszkowicz M., Fichna J., Rowe B.H., Porada E., Vincent R., Madsen K., Ghosh S., Storr M. (2012). Non-Specific Abdominal Pain and Air Pollution: A Novel Association. PLoS ONE.

[40] Lucht S.A., Hennig F., Matthiessen C., Ohlwein S., Icks A., Moebus S., Jöckel K.-H., Jakobs H., Hoffmann B. (2018). Air Pollution and Glucose Metabolism: An Analysis in Non-Diabetic Participants of the Heinz Nixdorf Recall Study. Environ. Health Perspect..

[41] Montnémery P., Popovic M., Andersson M., Greiff L., Nyberg P., Löfdahl C.-G., Svensson C., Persson C. (2003). Influence of heavy traffic, city dwelling and socio-economic status on nasal symptoms assessed in a postal population survey. Respir. Med..

[42] Pfeffer P.E., Donaldson G.C., Mackay A.J., Wedzicha J. (2019). Increased Chronic Obstructive Pulmonary Disease Exacerbations of Likely Viral Etiology Follow Elevated Ambient Nitrogen Oxides. Am. J. Respir. Crit. Care Med..

[43] Lebowitz M.D. (1998). Air Pollution and Hospital Admissions for Cardiovascular Disease. Epidemiology.

[44] Szyszkowicz M. (2007). Air Pollution and Emergency Department Visits for Ischemic Heart Disease in Montreal, Canada. Int. J. Occup. Med. Environ. Health.

[45] Szyszkowicz M. (2008). Ambient Air Pollution and Daily Emergency Department Visits for Headache in Ottawa, Canada. Headache J. Head Face Pain.

[46] Szyszkowicz M. (2008). Air Pollution and Daily Emergency Department Visits for Headache in Montreal, Canada. Headache J. Head Face Pain.

[47] Szyszkowicz M., Shutt R., Kousha T., Rowe B. (2014). Air pollution and emergency department visits for epistaxis. Clin. Otolaryngol..

[48] Szyszkowicz M., Kousha T., Kingsbury M., Colman I. (2016). Air Pollution and Emergency Department Visits for Depression: A Multicity Case-Crossover Study. Environ. Health Insights.

[49] Szyszkowicz M., Kousha T., Castner J. (2016). Air Pollution and Emergency Department Visits for Conjunctivitis: A Case—Crossover Case Study. Int. J. Occup. Med. Environ. Health.

[50] Szyszkowicz M., Kousha T., Castner J., Dales R. (2018). Air pollution and emergency department visits for respiratory diseases: A multi-city case crossover study. Environ. Res..

[51] Szyszkowicz M., Zemek R., Colman I., Gardner W., Kousha T., Smith-Doiron M. (2020). Air Pollution and Emergency Department Visits for Mental Disorders among Youth. Int. J. Environ. Res. Public Health.

[52] Vencloviene J., Grazuleviciene R., Babarskiene R., Dėdelė A., Grazulevicius T. (2011). Short-term nitrogen dioxide exposure and geomagnetic activity interaction: Contribution to emergency hospitalization for acute coronary syndrome. Int. J. Environ. Health Res..

[53] Yang Q., Chen Y., Krewski D., Burnett R.T., Shi Y., McGrail K.M. (2005). Effect of short-term exposure to low levels of gaseous pollutants on chronic obstructive pulmonary disease hospitalizations. Environ. Res..

[54] Yu O., Sheppard L., Lumley T., Koenig J.Q., Shapiro G.G. (2000). Effects of ambient air pollution on symptoms of asthma in Seattle-area children enrolled in the CAMP study. Environ. Health Perspect..

[55] Chen R., Pan G., Zhang Y., Xu Q., Zeng G., Xu X., Chen B., Kan H. (2011). Ambient carbon monoxide and daily mortality in three Chinese cities: The China Air Pollution and Health Effects Study (CAPES). Sci. Total. Environ..

[56] Bai L., Su X., Zhao D., Zhang Y., Cheng Q., Zhang H., Wang S., Xie M., Su H. (2018). Exposure to traffic-related air pollution and acute bronchitis in children: Season and age as modifiers. J. Epidemiol. Community Health.

[57] Guo B., Chen F., Deng Y., Zhang H., Qiao X., Qiao Z., Ji K., Zeng J., Luo B., Zhang W. (2018). Using rush hour and daytime exposure indicators to estimate the short-term mortality effects of air pollution: A case study in the Sichuan Basin, China. Environ. Pollut..

[58] Joseph P.M., Weiner M.G. (2002). Visits to Physicians after the Oxygenation of Gasoline in Philadelphia. Arch. Environ. Health Int. J..

[59] Kan H., Chen B., Zhao N., London S.J., Song G., Chen G., Zhang Y., Jiang L. (2010). Public Health and Air Pollution in Asia (PAPA): Coordinated Studies of Short-term Exposure to Air Pollution and Daily Mortality in Four Cities. Part 1: A Time-Series Study of Ambient Air Pollution and Daily Mortality in Shanghai, China; 2010. Res. Rep. Health Eff. Inst..

[60] Li P., Xin J., Wang Y., Wang S., Li G., Pan X., Liu Z., Wang L. (2013). The acute effects of fine particles on respiratory mortality and morbidity in Beijing, 2004–2009. Environ. Sci. Pollut. Res..

[61] Liu Y., Yan Z., Dong C. (2016). Health implications of improved air quality from Beijing’s driving restriction policy. Environ. Pollut..

[62] Meister K., Johansson C., Forsberg B. (2012). Estimated Short-Term Effects of Coarse Particles on Daily Mortality in Stockholm, Sweden. Environ. Health Perspect..

[63] Szyszkowicz M., Thomson E., Colman I., Rowe B.H. (2018). Ambient air pollution exposure and emergency department visits for substance abuse. PLoS ONE.

[64] Wei F., Wu M., Qian S., Li D., Jin M., Wang J., Shui L., Lin H., Tang M., Chen K. (2020). Association between short-term exposure to ambient air pollution and hospital visits for depression in China. Sci. Total. Environ..

[65] Wichmann J., Sjöberg K., Tang L., Haeger-Eugensson M., Rosengren A., Andersson E.M., Barregard L., Sallsten G. (2014). The effect of secondary inorganic aerosols, soot and the geographical origin of air mass on acute myocardial infarction hospitalisations in Gothenburg, Sweden during 1985–2010: A case-crossover study. Environ. Health.

[66] Wu Q., Xu Z., Dan Y.-L., Cheng J., Zhao C.-N., Mao Y.-M., Xiang K., Hu Y.-Q., He Y.-S., Pan H.-F. (2021). Association between traffic-related air pollution and hospital readmissions for rheumatoid arthritis in Hefei, China: A time-series study. Environ. Pollut..

[67] Zhang Y., Huang W., London S., Song G., Chen G., Jiang L., Zhao N., Chen B., Kan H. (2006). Ozone and Daily Mortality in Shanghai, China. Environ. Health Perspect..

[68] Evans K.A., Hopke P.K., Utell M.J., Kane C., Thurston S.W., Ling F.S., Chalupa D., Rich D.Q. (2016). Triggering of ST-elevation myocardial infarction by ambient wood smoke and other particulate and gaseous pollutants. J. Expo. Sci. Environ. Epidemiol..

[69] Sacks J.D., Ito K., Wilson W.E., Neas L.M. (2012). Impact of Covariate Models on the Assessment of the Air Pollution-Mortality Association in a Single- and Multipollutant Context. Am. J. Epidemiol..

[70] Szyszkowicz M. (2007). Air Pollution and Emergency Department Visits for Depression in Edmonton, Canada. Int. J. Occup. Med. Environ. Health.

[71] Szyszkowicz M. (2008). Ambient Air Pollution and Daily Emergency Department Visits for Ischemic Stroke in Edmonton, Canada. Int. J. Occup. Med. Environ. Health.

[72] Szyszkowicz M., Stieb D.M., Rowe B.H. (2009). Air pollution and daily ED visits for migraine and headache in Edmonton, Canada. Am. J. Emerg. Med..

[73] Szyszkowicz M., Willey J.B., Grafstein E., Rowe B.H., Colman I. (2010). Air Pollution and Emergency Department Visits for Suicide Attempts in Vancouver, Canada. Environ. Health Insights.

[74] Szyszkowicz M., Rowe B. (2010). Air pollution and emergency department visits for chest pain and weakness in Edmonton, Canada. Int. J. Occup. Med. Environ. Health.

[75] Zeka A., Zanobetti A., Schwartz J. (2005). Short term effects of particulate matter on cause specific mortality: Effects of lags and modification by city characteristics. Occup. Environ. Med..

[76] Zhou J., Ito K., Lall R., Lippmann M., Thurston G. (2011). Time-Series Analysis of Mortality Effects of Fine Particulate Matter Components in Detroit and Seattle. Environ. Health Perspect..

[77] Anderson H.R., de Leon A.P., Bland J.M., Bower J.S., Strachan D.P. (1996). Air pollution and daily mortality in London: 1987–1992. BMJ.

[78] Anderson H.R., Bremner S.A., Atkinson R.W., Harrison R.M., Walters S. (2001). Particulate matter and daily mortality and hospital admissions in the west midlands conurbation of the United Kingdom: Associations with fine and coarse particles, black smoke and sulphate. Occup. Environ. Med..

[79] Finnbjornsdottir R.G., Oudin A., Elvarsson B.T., Gislason T., Rafnsson V. (2015). Hydrogen sulfide and traffic-related air pollutants in association with increased mortality: A case-crossover study in Reykjavik, Iceland. BMJ Open.

[80] Hoek G., Brunekreef B., Verhoeff A., Van Wijnen J., Fischer P. (2000). Daily Mortality and Air Pollution in the Netherlands. J. Air Waste Manag. Assoc..

[81] Kaplan G.G., Dixon E., Panaccione R., Fong A., Chen L., Szyszkowicz M., Wheeler A., MacLean A., Buie W.D., Leung T. (2009). Effect of ambient air pollution on the incidence of appendicitis. Can. Med. Assoc. J..

[82] Lanki T., Pekkanen J., Aalto P., Elosua R., Berglind N., D’Ippoliti D., Kulmala M., Nyberg F., Peters A., Picciotto S. (2006). Associations of traffic related air pollutants with hospitalisation for first acute myocardial infarction: The HEAPSS study. Occup. Environ. Med..

[83] Pintarić S., Bodrožić-Džakic T., Pintarić H., Rusan Z., Ljubičić S. (2012). Effects of nitrogen dioxide and meteorological conditions on the number of patients presenting to emergency department. Acta Clin. Croat.

[84] Szyszkowicz M. (2008). Ambient Air Pollution and Daily Emergency Department Visits for Asthma in Edmonton, Canada. Int. J. Occup. Med. Environ. Health.

[85] Villeneuve P.J., Chen L., Stieb D., Rowe B.H. (2006). Associations between outdoor air pollution and emergency department visits for stroke in Edmonton, Canada. Eur. J. Epidemiol..

[86] Zemek R., Szyszkowicz M., Rowe B.H. (2010). Air Pollution and Emergency Department Visits for Otitis Media: A Case-Crossover Study in Edmonton, Canada. Environ. Health Perspect..

[87] Croft D.P., Cameron S.J., Morrell C.N., Lowenstein C.J., Ling F., Zareba W., Hopke P.K., Utell M.J., Thurston S.W., Thevenet-Morrison K. (2017). Associations between ambient wood smoke and other particulate pollutants and biomarkers of systemic inflammation, coagulation and thrombosis in cardiac patients. Environ. Res..

[88] Gordian E.M., Özkaynak H., Xue J., Morris S.S., Spengler J.D., Gordian M.E., Ozkaynak H., Xue J., Morris S.S., Spengler J.D. (2019). Particulate Air Pollution and Respiratory Disease in Anchorage, Alaska. Environ. Health Perspect..

[89] Keiding L.M., Rindel A.K., Kronborg D. (1995). Respiratory Illnesses in Children and Air Pollution in Copenhagen. Arch. Environ. Health Int. J..

[90] Kraus U., Breitner S., Schnelle-Kreis J., Cyrys J., Lanki T., Rückerl R., Schneider A., Brüske I., Gu J., Devlin R. (2011). Particle-associated organic compounds and symptoms in myocardial infarction survivors. Inhal. Toxicol..

[91] Liu L., Ruddy T., Dalipaj M., Poon R., Szyszkowicz M., You H., Dales R.E., Wheeler A.J. (2009). Effects of Indoor, Outdoor, and Personal Exposure to Particulate Air Pollution on Cardiovascular Physiology and Systemic Mediators in Seniors. J. Occup. Environ. Med..

[92] Stieb D.M., Shutt R., Kauri L., Roth G., Szyszkowicz M., Dobbin N.A., Chen L., Rigden M., Van Ryswyk K., Kulka R. (2018). Cardiorespiratory Effects of Air Pollution in a Panel Study of Winter Outdoor Physical Activity in Older Adults. J. Occup. Environ. Med..

[93] van der Zee S., Hoek G., Boezen H.M., Schouten J.P., van Wijnen J.H., Brunekreef B. (1999). Acute effects of urban air pollution on respiratory health of children with and without chronic respiratory symptoms. Occup. Environ. Med..

[94] Zhao Z., Zhang Z., Wang Z., Ferm M., Liang Y., Norbäck D. (2008). Asthmatic Symptoms among Pupils in Relation to Winter Indoor and Outdoor Air Pollution in Schools in Taiyuan, China. Environ. Health Perspect..

[95] Cui Y., Ai S., Liu Y., Qian Z.M., Wang C., Sun J., Sun X., Zhang S., Syberg K.M., Howard S. (2020). Hourly associations between ambient temperature and emergency ambulance calls in one central Chinese city: Call for an immediate emergency plan. Sci. Total Environ..

[96] Kloog I., Melly S.J., Coull B.A., Nordio F., Schwartz J.D. (2015). Using Satellite-Based Spatiotemporal Resolved Air Temperature Exposure to Study the Association between Ambient Air Temperature and Birth Outcomes in Massachusetts. Environ. Health Perspect..

[97] Chen K., Yang H.B., Ma Z., Bi J., Huang L. (2013). Influence of temperature to the short-term effects of various ozone metrics on daily mortality in Suzhou, China. Atmos. Environ..

[98] Meng X., Zhang Y., Zhao Z., Duan X., Xu X., Kan H. (2012). Temperature modifies the acute effect of particulate air pollution on mortality in eight Chinese cities. Sci. Total Environ..

[99] Li G., Zhou M., Cai Y., Zhang Y., Pan X. (2011). Does temperature enhance acute mortality effects of ambient particle pollution in Tianjin City, China. Sci. Total Environ..

[100] Wu S., Deng F., Liu Y., Shima M., Niu J., Huang Q., Guo X. (2013). Temperature, traffic-related air pollution, and heart rate variability in a panel of healthy adults. Environ. Res..

[101] Díaz J., López-Bueno J.A., López-Ossorio J., Gónzález J., Sánchez F., Linares C. (2020). Short-term effects of traffic noise on suicides and emergency hospital admissions due to anxiety and depression in Madrid (Spain). Sci. Total Environ..

[102] Ghada W., Estrella N., Pfoerringer D., Kanz K.-G., Bogner-Flatz V., Ankerst D.P., Menzel A. (2021). Effects of weather, air pollution and Oktoberfest on ambulance-transported emergency department admissions in Munich, Germany. Sci. Total Environ..

[103] Sangkharat K., Mahmood M.A., Thornes J.E., Fisher P.A., Pope F.D. (2020). Impact of extreme temperatures on ambulance dispatches in London, UK. Environ. Res..

[104] Roberts S. (2004). Interactions between particulate air pollution and temperature in air pollution mortality time series studies. Environ. Res..

[105] Bergmann S., Li B., Pilot E., Chen R., Wang B., Yang J. (2020). Effect modification of the short-term effects of air pollution on morbidity by season: A systematic review and meta-analysis. Sci. Total Environ..

[106] Kug J.-S., Jeong J.-H., Jang Y.-S., Kim B.-M., Folland C.K., Min S.-K., Son S.-W. (2015). Two distinct influences of Arctic warming on cold winters over North America and East Asia. Nat. Geosci..

[107] Nasa Earth Data Extremely Cold Texas in February 2021—One of the Coldest Februarys in Four Decades: The Story Told with NLDAS-2 Data. https://disc.sci.gsfc.nasa.gov/information/news?title=Extremely%20cold%20Texas%20in%20February%202021%20-%20one%20of%20the%20coldest%20Februarys%20in%20four%20decades:%20%20The%20story%20told%20with%20NLDAS-2%20data.

[108] Chan T.W., Meloche E., Kubsh J., Brezny R., Rosenblatt D., Rideout G. (2013). Impact of Ambient Temperature on Gaseous and Particle Emissions from a Direct Injection Gasoline Vehicle and its Implications on Particle Filtration. SAE Int. J. Fuels Lubr..

[109] Ko J., Jin D., Jang W., Myung C.-L., Kwon S., Park S. (2017). Comparative investigation of NOx emission characteristics from a Euro 6-compliant diesel passenger car over the NEDC and WLTC at various ambient temperatures. Appl. Energy.

[110] Suarez-Bertoa R., Astorga C. (2018). Impact of cold temperature on Euro 6 passenger car emissions. Environ. Pollut..

