# Supplementary material for: Cold Climate Impact on Air-Pollution-Related Health Outcomes: A Scoping Review
Source: Int J Environ Res Public Health. 2022 Jan 28;19(3):1473. doi: 10.3390/ijerph19031473 (PMC8835073; doi:10.3390/ijerph19031473)
Supplement: Supplementary file 1 [file ijerph-19-01473-s001.zip › ijerph-1532858-supplementary.pdf]

Review

Cold Climate Impact on Air-Pollution-Related Health Outcomes: A Scoping Review

Wine et al.

Supplementary Material

Table S1. Details of all reviewed papers.

(Abbreviations: CARD= cardiovascular disease, RESP= respiratory disease, MENT= mental disease, NEURO=neurologic disease, DIS= participants with pre-existing disease, CP=criteria pollutants, BS=black smoke, BC=black carbon)

Study ID	Study focus	Country/city	Years studied	Population	Health outcome or biomarker of disease	Pollutants studied	Pollution source and assessment	Season studied	Temperature < -7ºC (yes/no)	Study design and methodology	lag>1 day (yes/no)	Reported confounders	Pollutant associated	Main findings
Anderson 1996	Pollution and health	UK/Greater London	1987–1992	All population	Mortality	CP: O_3, NO_2, SO_2 Other: BS	Traffic emissions, monitoring stations	Summer and winter/cold and warm seasons and all year	No	Time series	Yes	Temperature, humidity, secular trends, seasonal and other cyclical factors, day of the week, holidays, an influenza epidemic	O_3, BS. smaller but still significant for NO_2 and SO_2	O_3 levels (same day) were associated with a significant increase in all-cause, cardiovascular, and respiratory mortality; the effects were more significant in the warm season and independent of other pollutants' impact. Black smoke concentrations on the previous day were significantly associated with all-cause mortality, and this effect was also greater in the warm season. Significant but smaller and less consistent effects were also observed for NO_2 and SO_2.
Anderson 2001	Pollution and health	UK	1994–1996	All population	Mortality/CARD/RESP	CP: PM_{10}, $PM_{2.5}$, NO_2, O_3, SO_2, CO Other: $PM_{2.5}$-10, BS, SO_4,	Source unspecified, monitoring stations	All year	No	Time series	Yes	Temperature and humidity, long term time trends, seasonal patterns, influenza epidemics, effects of a day of the week, and holidays	PM_{10}, $PM_{2.5}$ BS, SO_2, O_3	All-cause mortality was not associated with gaseous or particulate air pollutants in the all-year analysis. Analysis of admissions by age found evidence for various associations—notably between PM_{10}, $PM_{2.5}$, black smoke, SO_2, and ozone (negative) and respiratory admissions in the 0–14 age group. All measures of particles apart from $PM_{2.5}$–10 showed significant positive effects of the warm season
Andersson 2011	Pollution and health	Sweden/Lulea	2006	Children	RESP childhood asthma, wheeze, and allergic sensitization	(Setting) CP: NO_2, PM_{10}	Traffic emissions, proximity to daily traffic	All year (unclear when tests were done)	Yes	Proximity model	No	Meteorological factors, parental smoking, parental asthma, home dampness, sex, and socio-economic status. In the stratified analyses, the results were adjusted for parental asthma.	NA (traffic density)	proximity to heavy vehicle traffic was associated with current wheeze and asthma.
Bai 2018	Pollution and health	China/Hefei	2015–2016	Children	RESP acute bronchitis	CP: NO_2, $PM_{2.5}$, CO	Traffic emissions, monitoring networks	Summer and winter/cold and warm	No	Time series	Yes	Long-term trends, seasonal patterns	NO_2, $PM_{2.5}$, CO	NO_2, $PM_{2.5}$ and CO significantly increased the daily hospital visits for childhood acute

								seasons and all year						bronchitis in the cold season.
Bremner 1999	Pollution and health	UK/London	1992–1994	All population	Mortality	CP: PM_{10}, NO_2, SO_2, CO, O_3 Other: BC	Traffic emission, monitoring stations	Summer and winter/cold and warm seasons and all year	No	Poisson regression	No	Meteorology, trend, seasonality, calendar effects, deaths from influenza, and serial correlation.	PM_{10}, NO_2, O_3, BS	O_3 had a significant association with all-cause mortality. PM_{10} had the strongest association with respiratory mortality. NO_2, SO_2, CO, O_3, BC had associations with cardiovascular death but not PM_{10}.
Carlsen 2013	Pollution and health	Iceland/Reykjavik	2003–2009	Adult	CARD/RESP	CP: O_3, NO_2, PM_{10}	Traffic emission, monitoring stations	All year	Yes	Time series	Yes	Meteorological variables, day of the week, public holidays, and time trend with splines	O_3 and NO_2 but not $PM_{2.5}$	Daily emergency hospital visits increased with an increase in O_3. An increase in NO_2 increased hospital visits for females and the elderly. There were no associations with PM_{10}.
Chen 2011	Pollution and health	China/Shanghai, Anshan, and Taiyuan	2004–2008	ALL	Mortality	CP: CO, PM_{10}, SO_2, NO_2	Combination of sources, monitoring stations	All year	No	Time series	Yes	Weather	CO	Significant associations of CO with both total non-accidental and cardiovascular mortality were observed.
Chen 2013	Temperature and pollution combined effect and health	China/Suzhou	2008–2009	All population	Mortality	CP: O_3, PM_{10}, NO_2, SO_2	Source unspecified, monitoring stations	Summer and winter/cold and warm seasons and all year	No	Poisson generalized additive model	No	Co pollutants: PM_{10}, NO_2, SO_2	O_3	Cold season and low daily average temperatures significantly modified the acute effects of ozone on daily mortality.
Cheng 2012	Temperature and pollution combined impact and health	China/Shanghai	2001–2004	All population	Mortality	CP: PM_{10}, SO_2, NO_2, O_3	Source unspecified, monitoring stations	All year	No	Time-series	No	Mean temperature and relative humidity	PM_{10}, O_3	There was a statistically significant interaction between PM_{10}/O_3 and extremely low temperatures for total nonaccidental and cause-specific mortality. There was no significant interaction between SO_2 and NO_2.
Chimonas 2007	Pollution and health	USA/Anchorage, Alaska	1999–2003	Children	RESP Asthma, Lower respiratory illness	CP: PM_{10}, $PM_{2.5}$	Combination of sources (geologic, traffic) monitoring stations	All year	Yes	Time-series	Yes (whole week analysis)	Temperature, season year, weekends, wind speed, and precipitation	PM_{10} but not with $PM_{2.5}$	PM_{10} levels associated with outpatients' visits for asthma. $PM_{2.5}$ was not associated with any outcome.
Croft 2017	Pollution and health	USA/Rochester NY	2011–2013	Adults	Biomarkers of inflammation	Other: Delta-C, BC ultrafine particles (10–100 nm; UFP), accumulation mode (100–500 nm; AMP), and fine particles (< 2.5 μm; $PM_{2.5}$)	Combination of sources, monitoring stations and proximity	Winter/cold season only	Yes	Linear regression models	Yes	Temperature, humidity	Delta-C, $PM_{2.5}$, BC UFP	Delta-C concentration was associated with an increase in fibrinogen levels and MPO levels. Increases in $PM_{2.5}$, BC, UFP, and AMP concentrations were generally associated with increased CRP and fibrinogen, but not PF4, D-dimer, vWF, or P-selectin.
Cui 2020	Temperature/season and health (pollutant were adjusted)	China/Luoyang	2014–2016	All population	CARD/RESP/All-cause ambulance dispatch	CP: O_3, $PM_{2.5}$, NO_2 Other: $PM_{<2.5}$,	Source unspecified, monitoring stations	Summer and winter/cold and warm seasons and all year	Yes	Distributed nonlinear lag model (DLNM)	Yes	O_3, long term and seasonal trends	pollutants were adjusted for	Extreme cold temperatures were associated with all-cause, cardiovascular, and respiratory morbidity. Extreme hot temperatures were associated with all-cause and cardiovascular morbidity.
Dales 2020	Pollution and health	Canada/Ontario	2007–2012	DIS patients with ICD implant	CARD cardiac arrhythmia	CP: SO_2, O_3, NO_2, $PM_{2.5}$	Source unspecified, monitoring stations	Summer and winter/cold and warm seasons and all year	Yes	Case cross over	Yes	Daily mean temperature, barometric pressure and relative humidity, predictors of ICD discharge (*e.g.*, smoking, heart disease)	no association found	There is no evidence that the ambient air pollution concentrations observed in this study were a risk factor for potentially fatal cardiac arrhythmias in patients with ICDs.
Díaz 2020	Temperature/season and health (pollutant tested no effect found)	Spain/Madrid	2001–2013	All population	MENT anxiety, depression, suicide	CP: NO_2, PM_{10}, $PM_{2.5}$, O_3	Combination of sources (air and noise), monitoring stations	Summer and winter/cold and warm seasons and all year	No	Ecological longitudinal study	Yes	none	no association with pollutants was found	No association was found between pollutants noise and temp with mental diseases. However, an association was found between cold temperatures and anxiety.

Dockery 2005	Pollution and health	USA/Boston metropolitan, MA	1995–2002	DIS cardiac patients with ventricular arrhythmias and ICD devices	CARD arrhythmia	CP: $PM_{2.5}$, NO_2, CO, SO_2, O_3 Other: BC, SO_4, UFP, Sulfate	Source unspecified, monitoring stations	All year	yes	Logistic regression	Yes	Temperature, season, relative humidity, day of the week, and patient.	SO_2, $PM_{2.5}$, CO, NO_2	Significant associations of SVA with SO_2. VA and SVAs had positive associations with $PM_{2.5}$, CO, SO_2.
Evans 2017	Pollution and health	USA/Rochester NY	2007–2012	DIS cardiac patients treated at the Cardiac Catheterization Laboratory	CARD STEMI: ST-elevation myocardial infarction	CP: $PM_{2.5}$, SO_2, NO_2, O_3, CO Other: Delta-C, BC	Combination of sources, monitoring stations	Summer and winter/cold and warm seasons and all year	yes	Time-stratified case-crossover design	Yes	Weather conditions, age, chronic comorbidities, and long-term smoking history is controlled by design, air pollution	O_3, CO in cold $PM_{2.5}$in warm	Odds of STEMIs were associated with $PM_{2.5}$ in the warm season and O_3 and CO in the cold season. No increased odds were observed for Delta-C.
Finnbjornsdottir 2015	Pollution and health	Iceland/Reykjavik	2003–2009	Adult	Mortality	CP: NO_2, O_3, PM_{10}, SO_2 Other: H_2S	Combination of sources, monitoring stations	Summer and winter/cold and warm seasons and all year	No	Time stratified case-crossover	Yes	Temperature, relative humidity, influenza season, traffic-related pollutants, and meteorological variables	H2S	increased concentrations of H2S were associated With daily all-natural causes of mortality. The IR% was statistically significant among males and the elderly during the summer season.
Fung 2005	Pollution and health	Canada/Windsor, ON	1995–2001	All population	CARD	CP: O_3, CO, SO_2, PM_{10}, $PM_{2.5}$, Other: NO, COH	Combination of sources, monitoring stations	All year (max and min temps ranges)	Yes	Time series	Yes	Weather variables, seasonal, and weekly cycles	SO_2 (included PM_{10} in the model	SO_2 had the strongest effect on cardiac hospitalization among the >65 age group. When particulate PM_{10} was included in the model, the contributing impact of SO_2 remained significant for the >65 age group for all three levels.
Ghada 2020	Temperature/season and health (pollutant tested no effect found)	Germany/Munich	2014–2018	All population	ED admissions	CP: PM_{10}, O_3, NO_2 Other: NO	Combination of sources, monitoring stations	Four seasons and all year	No	Normal linear models	Yes	None	PM_{10}, NO_2	ER admissions were associated with higher temperatures in the warmer seasons. And colder minimum temperatures in the winter.
Gordian 1996	Pollution and health	USA/Anchorage, AK	1992–1994	All population	RESP	CP: PM_{10}, CO	Combination of sources (volcanic, incomplete combustion), monitoring stations	Winter/cold season only for CO and all year for PM_{10}	Yes	Time series	Yes	Temperature, day of the week long-term cycles, shared seasonal or monthly cycles among outpatient visits and environmental variables	CO in winter, PM_{10} all year	An increase in PM_{10} increased visits for asthma and upper respiratory diseases all year. Winter CO was associated with bronchitis and respiratory illness.
Grady 2018	Pollution and health	USA/Boston, MA	2012–2014	DIS COPD patents	RESP: lipid peroxidation and oxidative DNA damage in individuals with COPD	Other: BC	Traffic emissions, monitoring stations and home micro-environment sampler	Four seasons and all year	Yes	Linear mixed-effects regression models	Yes	Temperature and outdoor humidity race, heart disease requiring treatment in the past ten years, diabetes and smoking history. Age, statin use, nonsteroidal anti-inflammatory medication use, percent-predicted post-bronchodilator forced expiratory volume in one second, and time of urine collection. Body mass index (BMI), diabetes, heart disease, season, urine creatinine	BC (adjusted for $PM_{2.5}$ and NO_2)	Positive effects of black carbon on 8-OHdG and MDA results were similar when adjusting for $PM_{2.5}$ and NO_2. However, effects were more significant for obese participants.
Guo 2018	Pollution and health	China/Sichuan	2014–2016	All population	Mortality	CP: $PM_{2.5}$, SO_2, O_3	Traffic emissions, monitoring stations	All year: four seasons, warm and cold	No	Generalized additive model	Yes	Daily mean temperature and relative humidity, indicators for the day of the week, calendar time	O_3	Significant associations between O_3 and non-accidental and cardiovascular mortality in both winter and spring. These findings were more precise if using rush hour concentrations vs. 24 h averages.
Hagen 2000	Pollution and health	Norway/Drammen	1994–1997	All population	ED/RESP total acute hospital admissions and resp diseases	CP: PM_{10}, NO_2, O_3, SO_2 Other: NO, benzene, toluene, and	Combination of sources, monitoring stations and tapered element	All year	Yes	Time series	Yes	Temperature and humidity, periodic trends	PM_{10}, benzene	The relative risk of hospitalization is associated with increased PM_{10} and benzene.

						formaldehyde	oscillating microbalance technique							
Hertel 2010	Pollution and health	Germany/ Ruhr area: Essen, Mu¨lheim, Bochum)	2000–2003	DIS Cardiovascular cohort	Biomarkers systemic inflammation	CP: $PM_{2.5}$, PM_{10}, Other: PN	Source unspecified, monitoring stations	All year	Yes	Generalized additive models	Yes	Meteorology, season, time trend, and personal characteristics	PN	A positive association between PN and hs-CRP could be observed only for single-day lags and averaged PN concentrations with higher estimates for longer averaging times.
Hoek 2000	Pollution and health	Netherlands/Amsterdam, Rotterdam, The Hague, Utrecht	1986–1994	All population	Mortality	CP: PM_{10}, O_3, NO_2, SO_2, CO Other: BS, $SO_{4/-2}$, NO_3	Source unspecified, monitoring stations	Summer and winter/cold and warm seasons and all year	Yes	Poisson regression	Yes	Ambient temperature and relative humidity, long-term and seasonal trends, influenza epidemics, day of the week, and holidays	PM_{10}, and BS, O_3, NO_2, SO_2, CO $SO_{4/-2}$, NO_3	Daily mortality was significantly associated with the concentration of all air pollutants (PM_{10}, BS, O_3, NO_2, SO_2, CO $SO_{4/-2}$, NO_3). The RRs for all contaminants were substantially more significant in the summer months than in the winter months.
Joseph 2002	Pollution and health	USA/Philadelphia, PA	1992–1997	All population	Other: a collection of symptoms	Other: MTBE (methyl tertiary butyl ether)	Traffic emissions, periods when oxygenated fuel was used	Summer and winter/cold and warm seasons and all year	Yes (seasonal lag)	Ecological study	Yes (seasonal lag)	Weather and criteria pollutants	MTBE	Increasing patterns of asthma, wheezing, and other symptoms visit statistics in the winter could not be explained by pollution and weather data. The hypothesis of increased sensitization to MTBE or its combustion products explains it perfectly.
Kan 2010	Pollution and health	China/Shanghai	2001–2004	All population	Mortality	CP: PM_{10}, SO_2, NO_2, O_3	Combination of sources, monitoring stations	Summer and winter/cold and warm seasons and all year	No	Time series	Yes	Temperature, time trends stratified by sex and age, education level	PM_{10}, SO_2, NO_2, O_3	Short-term exposure to outdoor air pollution (PM_{10}, SO_2, NO_2, and O_3) was associated with daily mortality in Shanghai. Air pollution and daily mortality were more pronounced in the cool season.
Kaplan 2009	Pollution and health	Canada/Calgary, AB	1999–2006	Adults	Other: Appendicitis	CP: CO, NO_2, SO_2, O_3, PM_{10}, $PM_{2.5}$	Source unspecified, monitoring stations	Four seasons and all year	Yes	Case cross over	Yes	Temperature and relative humidity, effects of age, sex, and season	O_3, SO_2, NO_2, CO, PM_{10}	Some cases of appendicitis may be triggered by short-term exposure to air pollution O_3, SO_2, NO_2, CO, PM_{10}. A significant effect of the air pollutants in the summer months among men was observed but not among women.
Kaplan 2012	Pollution and health	Canada/Edmonton, AB and Montreal, QC	1992–2002	All population	Other: abdominal pain	CP: CO, NO_2, SO_2, O_3, PM_{10}, $PM_{2.5}$	Source unspecified, monitoring stations	All year	Yes	Case crossover	Yes	Temperature and relative humidity	CO, NO_2 in Edmonton and CO, NO_2, SO_2, $PM_{2.5}$ in Montreal	Epidemiological and animal data suggest that short-term exposure to air pollution (CO, NO_2 in Edmonton and CO NO_2, SO_2, $PM_{2.5}$ in Montreal) may trigger non-specific abdominal pain in young individuals.
Keiding 1995	Pollution and health	Denmark/ Copenhagen	1991 (Jan-April)	Children	RESP	CP: CO, SO_2, NO_2, O_3, Other: NO, NO_x, BS	Combination of sources, monitoring stations	Winter/ cold season only	Yes	Regression analysis	Yes	Temperature, systematic effects, and time trends	nitric oxide and NOx	Nitric oxide and NOx were associated with children's respiratory illnesses.
Kloog 2015	Temperature/season and health (pollutants were adjusted)	USA/MA	2000–2008	Children	Other: Birth outcomes	CP: predicted $PM_{2.5}$ Other: traffic density	Traffic emissions, satellite remote sensing, proximity, and monitoring stations	All year	Yes	Linear and mixed logistic models	Yes (entire pregnancy)	Individual-level socioeconomic status, traffic density, $PM_{2.5}$, random intercept for census tract, and mother's health.	NA pollutants ($PM_{2.5}$) were adjusted for	During pregnancy, an increase in temperature was associated with lower birth weight and shorter gestational age.
Kraus 2011	Pollution and health	Germany/ Augsburg	2003–2004	DIS cardiac patients: myocardial infarction survivors	CARD	CP: $PM_{2.5}$, Other: particle number concentration (PNC), $PM_{2.5}$-bound hopanes, and polycyclic aromatic hydrocarbons (PAH)	Combination of sources, monitoring stations	Winter/ cold season only	Not sure	Marginal models based on generalized estimating equations (GEE)	Yes	Meteorological variables: air temperature and relative humidity, long-term time trend and	POC, $PM_{2.5}$ hopanes, PNC, PAH	The study showed an association between PM, particle-bound POC, and daily symptoms.
Lanki 2006	Pollution and health	Europe/Augsburg, Barcelona,	1993–1999	All population	CARD First acute myocardial infraction	CP: CO, NO_2, PM_{10}, O_3 Other:	Traffic emissions,	Summer and winter/c	Maybe, min. not specified	Generalized additive models	Yes	Apparent temperature (temperature	CO, PNC, NO_2	CO, NO_2, and PNC were associated with

		Helsinki, Rome, Stockholm				PNC (particulate number concentration a proxy for ultra-fine Particles)	monitoring stations	old and warm seasons and all year		with Poisson regression		and humidity), barometric pressure		AMI. Effects were more pronounced in the warm season.
Li 2011	Temperature and pollution combined impact and health	China/Tianjin	2007–2009	All population	Mortality	CP: PM_{10}, NO_2, SO_2	Source unspecified, monitoring stations	All year:	Yes	Time-series	Yes	Seasonality, day of the week, relative humidity, and public holidays	PM_{10}	PM_{10} effects were stronger on high-temperature level days than those on low-temperature level days. The interactions between PM_{10} and temperature were statistically significant on cardiovascular, cardiopulmonary, and IHD mortalities.
Li 2013	Pollution and health	China/Beijing	2004–2009	All population	Mortality/RESP short term	CP: $PM_{2.5}$	Source unspecified, monitoring stations and tapered element oscillating microbalance technique	Summer and winter/cold and warm seasons and all year	Yes	Generalized additive Poisson regression	Yes	Temperature, humidity, exposure lag (in days), holiday, day of the week, and long-term trends	$PM_{2.5}$	Increasing $PM_{2.5}$ concentration increased respiratory mortality, and morbidity remarkably rose. The most potent adverse effect for both outcomes was observed in winter.
Liu 2009	Pollution and health	Canada/Ottawa, ON	2007 (Feb-March)	Seniors	CARD/RESP changes in cardiovascular function and plasma mediators in seniors. blood pressure, heart rate, and brachial artery function, and determined plasma mediators of inflammation, vascular function, and oxidative stress	CP: $PM_{2.5}$, Other: BC	Traffic emissions, monitoring stations	Winter/cold season only	Yes	Mixed-effects models	No	Temperature and relative humidity	$PM_{2.5}$, BC	Increases in black carbon and $PM_{2.5}$ were associated with increased effects on cardiovascular function and blood mediators that modulate vascular systems in seniors.
Liu 2016	Pollution and health	China/Beijing	2007–2009	All population	RESP	CP: API air pollution index (PM_{10}, SO_2 NO_2)	Traffic emissions, monitoring stations	Summer and winter/cold and warm seasons and all year	Yes	Comparative research design with general additive models (GAM)	No	Temperature, relative humidity, wind speed, duration of sunshine, average pressure, and holidays	API	The health effects of the driving restriction policy were stronger in the cold season for females and residents above 65 years old.
Lucht 2018	Pollution and health	Germany/ Ruhr area: Bochum, Essen, and Mülheim	2000–2003, 2006–2008	All population	Biomarkers of Glucose metabolism	CP: NO_2, PM_{10}, $PM_{2.5}$, Other: PNam (accumulation mode particle number)	Source unspecified, dispersion model	All year	No	Mixed linear regression	Yes	Temperature, humidity, season, meteorology, and personal characteristics: age, nutrition index, smoking status, and BMI, time since last meal	$PM_{2.5}$ and PNam and PM_{10} but not NO_2	Medium-term PM and PNAM exposures were positively associated with glucose measures in nondiabetic adults.
Meister 2012	Pollution and health	Sweden/Stockholm	2000–2008	All population	Mortality	CP: CO, PM_{10} and $PM_{2.5}$, O_3 Other: $PM_{2.5-10}$	Combination of sources (traffic suspended soil, road salt and sand studded tires, exhaust), monitoring stations	Summer and winter/cold and warm seasons and all year	No	Additive Poisson regression models	No	Temperature and relative humidity, other pollutants,	$PM_{2.5-10}$, road dust	Increase in daily mortality associated with elevated urban background levels of $PM_{2.5}$–10in the winter.
Meng 2012	Temperature and pollution combined effect and health	China/Guangzhou, Hangzhou, Shanghai, Shenyang, Suzhou, Taiyuan, Tianjin, and Wuhan	2001–2008	All population	Mortality	CP: PM_{10}	Source unspecified, monitoring stations	all year	Yes, for some of the cities	Time-series	Yes	Weather conditions	PM_{10}	The extremely high temperature increased the associations of PM_{10} with daily mortality.
Montgomery 2003	Pollution and health	Sweden/Southern part	1992	Adults	RESP nasal symptoms	(setting) CP: NO_2, SO_2 Other: Soot	Traffic emissions, heavy traffic as identified by respondents	All year	No	Multiple logistic regression	No	NA	NA (questionnaire)	A strong relation between geographical site and the prevalence of self-reported nasal symptoms was found
Pfeffer 2019	Pollution and health	UK/London	1996–2015	DIS patients with COPD	RESP COPD exacerbations	CP: PM_{10}, O_3 Other: NO_x	Combination of sources, monitoring stations	All year	No	Time series	Yes	Temperature, seasonality, and long-term trend	NOx	Higher levels of ambient NOx are associated with prolonged exacerbations of likely viral etiology.
Pintaric 2012	Pollution and health	Croatia/Zagreb	2008–2010	DIS patients with a cardiac referral to ED	CARD	CP: NO_2	Source unspecified, monitoring stations	Four seasons and all year	Yes	Multiple regression	No	None declared	NO_2	There was a positive correlation between the number of ED patients with cardiac referral diagnosis and increasing NO_2 concentrations in the air. Findings were most

														significant in the warm period
Sacks 2012	Pollution and health	USA/Philadelphia, PN	1992–1995	All population	Mortality	CP: $PM_{2.5}$, O_3, SO_2, NO_2, Other: trace elements: copper, zinc, bromine, lead, iron, silicon, calcium, manganese, nickel, vanadium, selenium, sulphur, and potassium.	Combination of sources, monitoring stations and filters	Summer and winter/cold and warm seasons and all year	Yes	Time series	No	Time and weather	$PM_{2.5}$ components SO_2 and NO_2	Factors associated with traffic and crustal material showed consistently positive associations in the warm season, while the coal combustion factor showed consistent positive associations in the cold season.
Sangkharat 2020	Temperature/season and health (pollutant tested no effect found)	UK/London	2010–2014	All population	CARD/RESP/Other ED	CP: $PM_{2.5}$ PM_{10}, O_3, NO_2, SO_2, CO Other: BC	Combination of sources, monitoring stations	Summer and winter/cold and warm seasons and all year	No	Time series	Yes	Air pollution	NA no association with pollutants was found	Significant associations were identified with extreme temperatures and ambulance dispatches. Significant associations were identified for respiratory categories and low temperatures. And significant associations of chest pain dispatch in high temperatures.
Schwartz 1996	Pollution and health	US/Spokane, WA	1988–1990	Seniors	RESP	CP: O_3, PM_{10}	Combination of sources (wood smoke, traffic), monitoring stations	All year	No	Poisson regression analysis	No	Temperature and relative humidity	PM_{10}, O_3	PM_{10} and O_3 were associated with an increased risk of respiratory hospital admissions. O_3 may be impacted by weather.
Stieb 2018	Pollution and health	Canada/Prince George, BC	2014, 2015 (Jan–April)	Adults	CARD/RESP cardiorespiratory effects	CP: CO, NO_2, SO_2, O_3, $PM_{2.5}$ and AQHI	Combination of sources, monitoring stations	Winter/cold season only	Yes	Time series	Yes	None declared	AQHI	Significant associations between air pollution and subclinical changes in daily measures of heart rate, blood pressure, and FEV1 among older adults exercising outdoors in winter in a small city characterized by moderate concentrations of both regional pollutants (ozone and $PM_{2.5}$) and traffic-related and industrial air pollutants (CO, NO_2, and SO_2). Heart rate exhibited the most consistent associations both with individual pollutants and the AQHI.
Szyszkowicz 2007	Pollution and health	Canada/Montreal, QC	1997-2002	DIS ischemic health diseases	CARD ischemic heart disease	CP: NO_2, CO	Source unspecified, monitoring stations	All year	Yes	Hierarchical clusters design	Yes	Temperature and relative humidity	NO_2, CO but no associations with SO_2 and O_3	Short-term effects of NO_2 and CO are associated significantly with daily ED visits for ischemic heart disease. For NO_2, the associations are stronger for patients aged over 64 years.
Szyszkowicz 2007	Pollution and health	Canada/Edmonton, AB	1992-2002	All population	MENT depression	CP: CO, NO_2, SO_2, O_3, PM_{10}, $PM_{2.5}$	Source unspecified, monitoring stations	Summer and winter/cold and warm seasons, and all year	Yes	Generalized linear mixed models (GLMM)	Yes	Temperature and relative humidity	CO NO_2, SO_2 O_3 in warm-season, PM_{10} $PM_{2.5}$ in cold	ED visits for depression are associated with exposure to ambient air pollution. CO, NO_2, SO_2, O_3 in warm-season PM_{10}, $PM_{2.5}$ in the cold season.
Szyszkowicz 2008	Pollution and health	Canada/QC Montreal	1997–2002	All population	NEURO headache	CP: O_3, NO_2, SO_2, CO, $PM_{2.5}$, PM_{10}	Source unspecified, monitoring stations	All year	Yes	Hierarchical clusters design	Yes	None declared	SO_2, NO_2, CO, $PM_{2.5}$	ED visits for headaches correlated to weather conditions and ambient air pollution: atmospheric pressure and exposure to SO_2, NO_2, CO, and $PM_{2.5}$.
Szyszkowicz 2008	Pollution and health	Canada/Edmonton, AB	1992–2002	All population	RESP ED visits for Asthma	CP: O_3, NO_2, SO_2, CO, $PM_{2.5}$, PM_{10}	Source unspecified, monitoring stations	Summer and winter/cold and warm seasons and all year	Yes	Generalized linear mixed model	Yes	Weather	NO_2, O_3	ED visits for asthma are associated with O_3 and NO_2 in the warm season.

Szyszkowicz 2008	Pollution and health	Canada/Edmonton, AB	1992–2002	All population	CARD ischemic stroke	CP: CO, NO_2, SO_2, O_3, PM_{10}, $PM_{2.5}$	Source unspecified, monitoring stations	Summer and winter/cold and warm seasons and all year	Yes	Generalized linear mixed models	Yes	Temperature and relative humidity	NO_2, SO_2 and O_3	NO_2, SO_2 and O_3 are significantly associated with ED visits for acute ischemic stroke. Associations differ by warm and cold seasons, lag, gender, and age.
Szyszkowicz 2008	Pollution and health	Canada/ON Ottawa	1992–2002	All population	NEURO headache	CP: SO_2, CO, NO_2,	Source unspecified, monitoring stations	All year	No	Time-series	Yes	Temperature, and relative humidity atmospheric pressure	SO_2, CO and NO_2	ED visits for headaches are related to ambient air pollution: SO_2, CO and NO_2.
Szyszkowicz 2009	Pollution and health	Canada/Edmonton, AB	1992–2002	All population	NEURO migraine and headache	CP: CO, NO_2, SO_2, O_3, PM_{10}, $PM_{2.5}$	Source unspecified, monitoring stations	Summer and winter/cold and warm seasons and all year	Yes	A Poisson model of counts hierarchically clustered	Yes	Temperature and relative humidity	$PM_{2.5}$ cold female, SO_2 and NO_2 warm male	There is an association between $PM_{2.5}$ in the cold season for females; SO_2 and NO_2 in the warm season for males. Preliminary evidence of an association between air pollution and ED visits for migraine and nonspecific headaches.
Szyszkowicz 2010	Pollution and health	Canada/Vancouver, BC	1999–2003	All population	MENT suicide attempts	CP: CO, NO_2, SO_2, O_3, PM_{10}, $PM_{2.5}$	Source unspecified, monitoring stations	Summer and winter/cold and warm seasons and all year	No	Case cross over	Yes	Temperature and relative humidity	CO, NO_2, SO_2, PM_{10} most significant for Male NO_2 in cold	There is a potential association between air pollution (CO, NO_2 SO_2, PM_{10}) and emergency department visits for suicide attempts. Associations differed by warm and cold seasons, lag gender and age.
Szyszkowicz 2010	Pollution and health	Canada/Edmonton, AB	1992–2002	All population	Other: chest pain and weakness	CP: CO, NO_2, SO_2, O_3, PM_{10}, $PM_{2.5}$	Source unspecified, monitoring stations	Summer and winter/cold and warm seasons and all year	Yes	generalized linear mixed models (GLMM)	Yes	Temperature and relative humidity	CO, NO_2, SO_2, PM_{10}	ED visits for chest pain and weakness are associated with exposure to ambient air pollution. Associations differ by cold and warm seasons, lag, age, and gender.
Szyszkowicz 2014	Pollution and health	Canada/Edmonton, AB	1992–2002	All population	Other: Epistaxis	CP: CO, NO_2, SO_2, O_3, PM_{10}, $PM_{2.5}$	Source unspecified, monitoring stations	All year	Yes	Case–crossover	Yes	Temperature and relative humidity	PM_{10}, O_3	There may be an association between O_3 and PM_{10} and ED visits for epistaxis.
Szyszkowicz 2016	Pollution and health	Canada/ON	2004–2011	All population	MENT depression	CP: NO_2, O_3, $PM_{2.5}$, SO_2	Source unspecified, monitoring stations	Summer and winter/cold and warm seasons an all year	Yes	Case-crossover	Yes	Temperature and relative humidity	O_3	A positive association between exposure to O_3 and ED visits for depression.
Szyszkowicz 2016	Pollution and health	Canada/Algoma, Halton, Hamilton, London, Ottawa, Peel, Toronto, Windsor, and York, ON	2004–2011	All population	Other: conjunctivitis	CP: NO_2 O_3 $PM_{2.5}$ SO_2	Combination of sources, monitoring stations	Summer and winter/cold and warm seasons and all year	Yes	Time-stratified case-crossover design	Yes	Temperature and relative humidity	NO_2, $PM_{2.5}$, for females and NO_2, O_3 $PM_{2.5}$ for males	Associations between levels of air pollution (NO_2, $PM_{2.5}$, for females and NO_2, O_3 $PM_{2.5}$ for males) and ED visits for conjunctivitis, with different temporal trends and strength of association by age, sex, and season.
Szyszkowicz 2018	Pollution and health	Canada/Algoma, Halton, Hamilton, Middlesex, Ottawa, Peel, Toronto, Essex, and York, ON	2004–2011	All population	RESP	CP: NO_2, O_3 $PM_{2.5}$, SO_2	Combination of sources, monitoring stations	Summer and winter/cold and warm seasons and all year	Yes	Case cross over	Yes	Temperature and relative humidity, and an influenza indicator	NO_2, $PM_{2.5}$, SO_2, O_3	Associations between short-term exposure to air pollution (NO_2, $PM_{2.5}$, SO_2, O_3) and increased risk of ED visits for upper and lower respiratory diseases in an environment where air pollutant concentrations are relatively low.
Szyszkowicz 2018	Pollution and health	Canada/Edmonton, AB	1992–2002	All population	MENT substance use	CP: CO, NO_2, SO_2, O_3, $PM_{2.5}$ and AQHI	Source unspecified, monitoring stations	Summer and winter/cold and warm seasons and all year	Yes	Time-stratified case-crossover design	Yes	Temperature and relative humidity	strongest CO NO_2 cold, positive results also with PM_{10} and $PM_{2.5}$	Even at low levels, increases in ambient CO, NO_2, and PMs are associated with increased hospital admissions for substance abuse. The strongest results were obtained in the cold period.
Szyszkowicz 2020	Pollution and health	Canada/Toronto, ON	2004–2005	Children	MENT mental disorders	CP: NO_2, O_3, $PM_{2.5}$	Combination of sources (traffic, noise), monitoring stations	All year	Yes	Time stratified case-crossover	Yes	Temperature	$PM_{2.5}$, NO_2, O_3	PMs and NO_2 are associated with an increased risk for ED visits for adolescents and young adults with diagnosed mental

														health disorders. O_3 was identified in one instance.
vanderZee 1999	Pollution and health	Netherlands/Rotterdam, Bodegraven-Reeuwijk, Amsterdam, Meppel, and Nunspeet	1992–1993 (winters)	Children	RESP	CP: PM_{10}, SO_2, NO_2, Other: BS, sulphate	Traffic emissions, monitoring stations	Winter/cold season only	Yes	Logistic regression	Yes	Temperature, time trends (generally in the order of weeks) in the prevalence of symptoms, use of bronchodilators, and decrements in PEF	PM_{10}, BS, sulphate	Significant associations were found between PM_{10}, black smoke, sulphate concentrations and the prevalence of lower respiratory tract symptoms and decrements in PEF.
Venclovienе 2011	Pollution and health	Lithuania	2004–2006	All population	CARD acute coronary syndrome	CP: NO_2	Traffic emissions, monitoring stations	All year	Yes	Case-crossover	Yes	Meteorological factors: air temperature, wind speed, relative humidity and barometric pressure, solar and geomagnetic activity seasonal variation, weekdays and	NO_2 as effect modification	Geomagnetic activity variations may increase the traffic-related air pollution effect on the acute coronary syndrome.
Villeneuve 2006	Pollution and health	Canada/Edmonton, AB	1992–2002	Seniors	CARD stroke	CP: CO, NO_2, SO_2, O_3, PM_{10} and $PM_{2.5}$	Traffic emissions, monitoring stations	Summer and winter/cold and warm seasons and all year	Yes	Time-stratified case-crossover study	Yes	Temperature and relative humidity	NO_2, CO	Elevated risks were observed between levels of air pollution and acute ischemic stroke in the warm season. The results from our multi-pollutant modelling implicate NO_2 and CO as the pollutants of primary concern.
Wei 2020	Pollution and health	China/Ningbo	2013–2018	All population	MENT depression	CP: CO, NO_2, SO_2, O_3, PM_{10}	Combination of sources, monitoring stations	Summer and winter/cold and warm seasons and all year	No	Quasi-Poisson regression models with generalized additive models (GAM)	Yes	Temperature, humidity, long-term trend, seasonality, day of week and holiday and other pollutants CO and NO_2	$PM_{2.5}$, PM_{10}, SO_2, CO, and NO_2	$PM_{2.5}$, PM_{10}, SO_2, CO, and NO_2 were positively correlated with hospital visits for depression. The associations were stronger in the elderly (≥65 years) and the cold season.
Wichmann 2014	Pollution and health	Sweden/Gothenburg	1985–2010	All population	CARD acute myocardial infarction	CP: PM_{10}, $PM_{2.5}$, NO_2, O_3 Other: PMion (sum of sulphate, nitrate, and ammonium) and soot). Pmrest (The difference between urban PM_{10} and rural PMion was a proxy for locally generated PM_{10}), NOx	Combination of sources, monitoring stations	Summer and winter/cold and warm seasons and all year	Yes	case-crossover	No	Temperature and relative humidity, public holidays	PM rest	In the cold period, locally generated PM and limited local dispersion affected AMI hospitalizations.
Wu 2013	Temperature and pollution combined effect and health	China/Beijing	2008–2009	Adults	CARD Cardiac autonomic function, heart rate variability	CP: $PM_{2.5}$, CO	Traffic emissions, portable spectrometer, CO measurer	Summer and winter/cold and warm seasons only	No	Time series	No	Age, time of day, log10-transformed heart rate and relative humidity, day of the year and gender, body mass index, years as a taxi driver, day of the week, status of cab windows and status of cab air-conditioner.	$PM_{2.5}$, CO	The temperature in both cold and warm seasons was associated with alterations in cardiac autonomic function in healthy adults in the context of traffic-related air pollution. $PM_{2.5}$ and CO modified these associations to various extents.
Wu 2021	Pollution and health	China/Hefei	2014–2018	DIS RA patients	Other: Rheumatoid arthritis	CP: CO, NO_2, O_3, PM_{10}, $PM_{2.5}$	Traffic emissions, monitoring stations	Summer and winter/cold and warm seasons and all year	No	Time series	Yes	$PM_{2.5}$, relative humidity, mean temperature, long-term trends, dummy variable of the day of week and holidays	$PM_{2.5}$ NO_2	$PM_{2.5}$ and NO_2 were associated with rheumatoid arthritis admissions in the cold season.
Yang 2005	Pollution and health	Canada/Vancouver, BC	1994–1998	Seniors	RESP: acute COPD	CP: CO, NO_2, SO_2, O_3, PM_{10}	Traffic emissions, monitoring stations	Four seasons and all year	Yes	Time series	Yes	Weather variables, seasonal and sub-seasonal fluctuations, non-Poisson dispersion	NO_2, CO but no association with SO_2 and O_3	NO_2 and CO were significantly associated with hospitalization for COPD. No significant association were identified between either SO_2 or ozone and COPD
Yu 2000	Pollution and health	US/Seattle, WA	1993–1995	Children	RESP Asthma	CP: CO, SO_2, $PM_{1.0}$ and AQHI	Source unspecified, monitoring stations	All year	No	Logistic regression a marginal approach [generalized estimating equations (GEE)	Yes	Dependent confounders (day of week, season, and temperature) subject-specific variables (age, race, sex,	CO, $PM_{1.0}$, PM_{10} no association with SO_2	There is an association between change in short-term air pollution levels, as indexed by PM1.0 and PM_{10} and CO, and the occurrence

												baseline height, and FEV1 PC20 concentration) and potential time-		of asthma symptoms among children in Seattle.
Zeka 2005	Pollution and health	USA/20 cities	1989–2000	All population	Mortality	CP: PM_{10}	Traffic emissions, monitoring stations	Summer and winter/cold and warm seasons	Yes	Case cross over	Yes	Temperature, and percentage of primary PM_{10} from traffic sources. the prevalence of central air conditioning, population density, standardized mortality rates, the proportion of elderly in each city,	PM_{10}	All-cause mortality increased with PM_{10} increase in both higher and lower temperatures. Heart disease mortality after two days of exposure and respiratory disease on all three days of exposure
Zemek 2010	Pollution and health	Canada/Edmonton, AB	1992–2002	Children	Other: Otitis media	CP: CO, PM_{10}, $PM_{2.5}$, SO_2, NO_2, O_3	Source unspecified, monitoring stations	Summer and winter/cold and warm seasons	Yes	Time-stratified case-crossover technique	Yes	Weather variables	NO_2, CO	ED visits for otitis media are associated with ambient air pollution: NO_2 and CO. The strongest associations were observed in the warmer months.
Zhang 2006	Pollution and health	China/Shanghai	2001–2004	All population	Mortality	CP: O_3, PM_{10}, NO_2 SO_2	Combination of sources (coal, traffic), monitoring stations	Summer and winter/cold and warm seasons and all year	No	Time series	Yes	Temperature, seasonality, long-term trends, and other pollutants: PM_{10}, SO_2, NO_2	O_3	O_3 pollution has stronger health effects in the cold than in the warm season in Shanghai.
Zhao 2008	Pollution and health	China/Taiyunan	2004	Children	RESP asthma	CP: SO_2, NO_2, O_3 Other: formaldehyde	Combination of sources (coal, traffic), monitoring stations	Winter/cold season only	No	Cross-sectional study	No	Age, sex, parental asthma or allergy, and home environmental factors (new painting, new floor material, new furniture, and ETS)	SO_2, NO_2, formaldehyde	Wheeze or daytime or nocturnal attacks of breathlessness were positively associated with SO_2, NO_2, or formaldehyde.
Zhou 2011	Pollution and health	USA/Detroit, MI and Seattle, WA	2002–2004	All population	Mortality	CP: $PM_{2.5}$, CO, NO_2, O_3 Other: $PM_{2.5}$ components	Combination of sources (mixture), monitoring stations	Summer and winter/cold and warm seasons and all year	Yes	Poisson regression and distributed lag models	Yes	Temperature and relative humidity, other gaseous pollutants	$PM_{2.5}$	The $PM_{2.5}$ components and gaseous pollutants associated with mortality in Detroit were mainly related to warm-season secondary aerosols and traffic markers. The component species most closely associated with mortality in Seattle included cold season traffic and other combustion sources, such as residual oil and wood burning. There were composition changes according to season and mixed sources

Table S2. The complete data on the pollutants tested in the papers reviewed (no pattern of specific pollutants tested at the same study was found):.

Criteria pollutants	CO, NO_2, SO_2, O_3, PM_{10}, $PM_{2.5}$, air quality health index (AQHI), air pollution index (API)
Other pollutants	Black carbon (BC), black smoke (BS), NO, NO_x, NO_3, SO_4, sulphate H_2S PAHs, COH, trace elements (copper, zinc, bromine, lead, iron, silicon, calcium, manganese, nickel, vanadium, selenium, sulphur, and potassium)<br>Volatile Organic compounds (VOCs): Formaldehyde, Benzene, Toluene, Methyl tert-butyl ether (MTBE)<br>Particles: PNC (particulate number concentration/UFP indicator) or UFP, Delta- C, $PM_{2.5\text{-}10}$, PN (particulate number), PNam (accumulation mode particle number), $PM_{2.5}$ bound hopanes, PMion (sum of sulphate nitrate and ammonium), PMrest (The difference between urban PM_{10} and rural; a proxy for locally generated PM_{10}), $PM_{2.5}$ components, $PM_{<2.5}$, AMP (accumulation mode)

Only Criteria pollutants were studied ($n = 45$)	One criteria pollutant	5	2(NO_2), 2(PM_{10}), ($PM_{2.5}$)
	Two criteria pollutants	5	(NO_2, CO), (PM_{10}, O_3), (PM_{10}, CO), (PM_{10}, $PM_{2.5}$), ($PM_{2.5}$, CO)
	Three criteria pollutants	7	(NO_2, $PM_{2.5}$, CO), (NO_2, $PM_{2.5}$, O_3), (NO_2, PM_{10}, SO_2), ($PM_{2.5}$, SO_2, O_3), (NO_2, SO_2, CO), (NO_2, PM_{10}, SO_2/API), (NO_2, PM_{10}, O_3)
	Four criteria pollutants	10	4(NO_2, $PM_{2.5}$, O_3, SO_2), 4(NO_2, PM_{10}, O_3, SO_2), (NO_2, CO, PM_{10}, SO_2), (NO_2, $PM_{2.5}$, PM_{10}, O_3)
	Five criteria pollutants	5	2(NO_2, CO, SO_2, O_3, PM_{10}), 2(NO_2, CO, SO_2, O_3, $PM_{2.5}$ and AQHI), (NO_2, PM_{10}, $PM_{2.5}$, CO, O_3)
	Six criteria pollutants	12	12(NO_2, PM_{10}, $PM_{2.5}$, CO, SO_2, O_3)
Criteria pollutants + other various pollutants were studied ($n = 26$)	One criteria pollutant	3	($PM_{2.5}$+BC),<br>($PM_{2.5}$+PNC, $PM_{2.5}$hopanes, PAH),<br>($PM_{2.5}$+traffic density)
	Two criteria pollutants	3	(PM_{10}, O_3+NOx), ($PM_{2.5}$, PM_{10}+PN), (CO, SO_2, AQHI +$PM_{1.0}$)
	Three criteria pollutants	6	(NO_2, O_3, SO_2+ BS), (NO_2, PM_{10}, SO_2+BS, sulfate), (NO_2, PM_{10}, O_3+NO), (NO_2, SO_2, O_3+formaldehyde), (NO_2, PM_{10}, $PM_{2.5}$+PNam), (NO_2, $PM_{2.5}$, O_3 +$PM_{<2.5}$)
	Four criteria pollutants	8	(NO_2, PM_{10}, CO, O_3 +PNC)<br>(NO_2, PM_{10}, $PM_{2.5}$, O_3 + PMion, Pmrest, NOx), (NO_2, PM_{10}, O_3, SO_2 +NO, benzene, toluene, and formaldehyde), (NO_2, $PM_{2.5}$, CO, O_3 + $PM_{2.5}$ components) (NO_2, $PM_{2.5}$, O_3, SO_2 + trace elements), (PM_{10}, $PM_{2.5}$, CO, O_3 +$PM_{2.5-10}$), (NO_2, CO, SO_2, O_3 + NO, NO_x, BS), (NO_2, O_3, PM_{10}, SO_2+H_2S)
	Five criteria pollutants	5	(PM_{10}, $PM_{2.5}$, O_3, CO, SO_2, + NO, COH), (NO_2, PM_{10}, SO_2, CO, O_3+ BC),<br>(NO_2, $PM_{2.5}$, CO, SO_2, O_3+ BC, SO_4, UFP, Sulfate), (NO_2, $PM_{2.5}$, SO_2, O_3, CO +Delta-C, BC), (NO_2, PM_{10}, O_3, SO_2, CO + BS, $SO_{4/-2}$, NO_3)
	Six criteria pollutants	2	(NO_2, PM_{10}, $PM_{2.5}$, CO, SO_2, O_3+$PM_{2.5-10}$, BS, SO_4), (NO_2, PM_{10}, $PM_{2.5}$, CO, SO_2, O_3+BC)
Only non-criteria pollutants were studied ($n = 3$)		3	(BC), (BC, Delta-C, UFP, AMP, $PM_{<2.5}$), (MTBE)
Pollutants were not studied, but levels were described as setting ($n = 2$)	2	2	(NO_2, SO_2 and Soot) (NO_2, PM_{10})

Table S3. The complete data on the papers which studied seasonal associations with health outcomes: types of health outcomes, pollutants associated, and temperature range indicated (no pattern identified).

	No Seasonal or Temperature Associations Indicated	Associations Stronger in Cold Seasons	Associations in Both Cold and Warm Seasons	Associations Stronger in Warm Seasons	Winter Data Only
Number of studies	27	12	9	10	8
Health outcomes	RESP (9), CARD (5) other (3) MENT (2) mortality (2) NEURO (2) biomarkers (2) CARD and RESP	Mortality (4) MENT (2) RESP (2) CARD (1) other (2) mortality and RESP (1)	Mortality (3), CARD (2), MENT (2), NEURO (1), other (1)	Mortality (3), other (2) mortality and CARD and RESP (1), CARD (3) RESP (1)	RESP (4), CARD and RESP (2) CARD (1), biomarkers of inflammation (1)

	and Stroke (1) ED and RESP (1)				
Pollutants associated	NO_2 (12) 44%, O_3 (8)30%, CO (8)30%, $PM_{2.5}$ (7)26%, PM_{10} (7)26%, SO_2 (6)22%, Other (6)22%: BC (1), benzene (1), PN (1), PNAM (1), NO_x (1), BS (1)	NO_2 (5) 41% $PM_{2.5}$ (5) 41% O_3(3)25% PM_{10}(3)25% CO (3)25% SO_2(2)16% Other (4)33%: $PM_{2.5-10}$ (1), PMrest (1), API (1), MTBE (1)	Warm: CO (1), NO_2(2), $PM_{2.5}$ (1), SO_2(2), O_3(2) Cold: PM_{10}(2), $PM_{2.5}$ (2), NO_2(1), SO_2(1), O_3(1) Both: NO_2(3), SO_2(3), PM_{10}(2), $PM_{2.5}$ (1)	NO_2(8)80%, CO (5)50%, O_3(4)40%, PM_{10}(3)30%, SO_2(3)30%, $PM_{2.5}$ (1)10%, Other (6) 60%): BS (2), H_2S (1), SO_4(1), NO_3(1), PNC (1)	$PM_{2.5}$ (3), PM_{10}(1), SO_2(1), NO_2(1), CO (1), AQHI (1), Others: BC (2), POC (1), hopanes (1) PNC (1) PAH (1), NO (1), BS (1), sulfate (1), formaldehyde (1), Delta-C (1), UFP (1)
Temperatures 0C to −7 °C	8	7	2	3+1(not specified)	2
Temperatures below −7 °C	19	5	7	6	6
